# Effects of exercise therapy in patients with acute low back pain: a systematic review of systematic reviews

**DOI:** 10.1186/s13643-020-01412-8

**Published:** 2020-08-14

**Authors:** Marc Karlsson, Anna Bergenheim, Maria E. H. Larsson, Lena Nordeman, Maurits van Tulder, Susanne Bernhardsson

**Affiliations:** 1 Region Skåne, Healthcare Centre Oxie, Malmö, Sweden; 2 Department of Health and Rehabilitation, Unit of Physiotherapy, The Sahlgrenska Academy, Institute of Neuroscience and Physiology, University of Gothenburg, Gothenburg, Sweden; 3 Region Västra Götaland, Närhälsan Uddevalla Rehabilitation, Uddevalla, Sweden; 4 Region Västra Götaland, Research and Development Primary Health Care, Kungsgatan 12, 6th floor, SE-412 19 Gothenburg, Sweden; 5 Department of Health Sciences, Faculty of Science, Amsterdam Movement Sciences, Vrije Universiteit Amsterdam, Amsterdam, the Netherlands; 6 Department Physiotherapy & Occupational Therapy, Aarhus University Hospital and Aarhus University, Aarhus, Denmark

**Keywords:** Exercise therapy, Acute low back pain, Systematic review, Evidence-based, GRADE

## Abstract

**Background:**

Acute low back pain is associated with pain and disability, but symptoms are often self-healing. The effectiveness of exercise therapy for acute low back pain remains uncertain with conflicting evidence from systematic reviews. The aim of this systematic review of systematic reviews was to assess the overall certainty of evidence for the effects of exercise therapy, compared with other interventions, on pain, disability, recurrence, and adverse effects in adult patients with acute low back pain.

**Methods:**

PubMed, the Cochrane library, CINAHL, PEDro, Open Grey, Web of Science, and PROSPERO were searched for systematic reviews of randomized controlled trials. Methodological quality was assessed independently by two authors using AMSTAR. Meta-analyses were performed if possible, using data from the original studies. Data for pain, disability, recurrence, and adverse effects were analyzed. Certainty of evidence was assessed using GRADE.

**Results:**

The searches retrieved 2602 records, of which 134 publications were selected for full-text screening. Twenty-four reviews were included, in which 21 randomized controlled trials (*n* = 2685) presented data for an acute population, related to 69 comparisons. Overlap was high, 76%, with a corrected covered area of 0.14. Methodological quality varied from low to high. Exercise therapy was categorized into general exercise therapy, stabilization exercise, and McKenzie therapy. No important difference in pain or disability was evident when exercise therapy was compared with sham ultrasound, nor for the comparators usual care, spinal manipulative therapy, advice to stay active, and educational booklet. Neither McKenzie therapy nor stabilization exercise yielded any important difference in effects compared with other types of exercise therapy. Certainty of evidence varied from very low to moderate.

**Conclusions:**

The findings suggest very low to moderate certainty of evidence that exercise therapy may result in little or no important difference in pain or disability, compared with other interventions, in adult patients with acute low back pain. A limitation of this systematic review is that some included reviews were of low quality. When implementing findings of this systematic review in clinical practice, patients’ preferences and the clinician’s expertise also should be considered, to determine if and when exercise therapy should be the intervention of choice.

**Systematic review registration:**

PROSPERO: CRD46146, available at: https://www.crd.york.ac.uk/PROSPERO/display_record.php?RecordID=46146.

## Background

Low back pain (LBP) affects approximately 70% of the adult population in the western world, at some point in their lives [1, 2]. The economic burden for society is high [1, 3], and LBP is ranked as one of the three most burdensome conditions in terms of years lived with disability [4]. In the acute phase of LBP, lasting up to 6 weeks, many individuals suffer considerable pain and disability. The prognosis for acute LBP is favorable. Most symptoms resolve within 6 weeks in 70–80% of affected patients, regardless of intervention or no intervention at all [5–8]. A small proportion, about 5%, suffer persistent or recurrent pain leading to prolonged disability, which may further abate recovery [6]. Many patients with acute LBP are managed in primary care settings and may visit a physiotherapist [3]. Physiotherapists offer many interventions for this patient group, of which one of the most widely used is exercise therapy [9–11]. This practice is concordant with clinical guidelines for the management of low back pain in primary care [12].

International, evidence-based, clinical practice guidelines exist that can aid the physiotherapist in choosing appropriate interventions for patients with acute LBP [13–15]. However, recommendations in various guidelines, produced in different countries, differ and are not always consistent with results from systematic reviews [13, 16–18]. This may seem odd since systematic reviews of randomized controlled trials (RCTs) provide top level evidence and should be the fundament for any high-quality evidence-based guideline [19]. However, in the development of clinical guidelines, evidence for effectiveness and safety of interventions are only two aspects that are considered. Other aspects such as costs, feasibility, patient preferences, and availability are usually also considered when translating the evidence into recommendations for clinical practice. A mix of populations (acute, sub-acute, and chronic LBP populations) and different definitions of duration are other factors that may explain why recommendations vary [13, 14, 16]. We know that intervention effects differ depending on whether the pain is acute, sub-acute, or chronic [17, 18, 20], and that acute pain differs from chronic pain [21].

The most uniform recommendation in international guidelines is that “first line care” for patients with acute LBP should be to give reassurance, advice to stay active in daily life, and, if necessary, pain medication [12, 13]. Exercise therapy, spinal manipulative therapy, mobilization, and acupuncture are other interventions recommended in some, but not all, clinical practice guidelines [12, 13, 16] and typically if first line care did not lead to improvement of symptoms. For these interventions, there are discrepancies regarding when, how, and whether they should be used in acute LBP [12, 13, 15, 22].

Within the umbrella term exercise therapy, several types of exercise therapy exist. From a physiotherapist perspective, the following types can be distinguished: McKenzie therapy, stabilization exercises (also called motor control exercise), strengthening (or resistance) exercises, stretching exercises, and aerobic exercises. These different types of exercise therapy differ in one important aspect; the hypothesized underpinning effect mechanism. Some have a rather solid theory (physiology), for example aerobic exercise, while others have a more conceptualized theory, for example McKenzie therapy, influenced by the persons that introduced that particular type of exercise therapy. Still, all fit within the umbrella term exercise therapy.

Despite guidelines recommending different interventions, several systematic reviews show that interventions for patients with acute LBP rarely yield clinically relevant effects compared with placebo treatment [17, 23, 24]. This seems to be the case not only concerning physiotherapeutic interventions but for pharmacological treatment as well [25]. Exercise therapy, frequently used in clinical physiotherapy practice [9–11], is no exception from this uncertainty of clinically relevant effect [7, 17, 26, 27]. Many systematic reviews conclude that exercise therapy is effective for patient with acute LBP, but that the evidence is inconclusive [20, 28]. Although systematic reviews may be well conducted, the often low methodological quality of many of the included studies reduces the confidence we may have in conclusions regarding exercise therapy and its clinically relevant effects [14].

The limitations and issues described imply a need to summarize and synthesize the findings from existing systematic reviews on exercise therapy, and to assess the overall certainty of evidence for effect of this common physiotherapeutic intervention for acute LBP. To the best of our knowledge, no systematic review of systematic reviews on this topic has been published. The aim of this systematic review of systematic reviews was to assess the overall certainty of evidence for the effects of exercise therapy provided by physiotherapists in comparison with other interventions, on pain, disability, recurrence, and adverse effects in adult patients with acute LBP.

## Methods

### Protocol and registration

We conducted this systematic review of systematic reviews according to a protocol registered in PROSPERO (CRD46146), available at: https://www.crd.york.ac.uk/PROSPERO/display_record.php?RecordID=46146. The protocol was not published in any peer-reviewed journal. The development of the protocol was guided by the Preferred Reporting Items for Systematic Review and Meta-Analysis Protocols (PRISMA-P) 2015 statement [29]. Conduct and reporting followed Smith et al.’s [30] methodological recommendations for conducting a systematic review of systematic reviews of healthcare interventions, the Cochrane Collaboration’s recommendations for conducting an overview of systematic reviews [31], Lunny et al.’s [32, 33] comprehensive publications with recommendation for conducting an overview, and the PRISMA statement [34] (Additional file 1).

### Eligibility criteria

Exercise therapy is defined as “a regimen or plan of physical activities designed and prescribed for specific therapeutic goals, with the purpose to restore normal musculoskeletal function or to reduce pain caused by diseases or injuries” [35]. In this systematic review, interventions were classified as exercise therapy if they could be carried out in physiotherapy and when no other intervention dominated the intervention. For example, the concept of McKenzie therapy involves repeated exercises following a directional preference, but also sometimes spinal mobilization or manipulation [36].

Inclusion criteria and a priori determined definitions for clinical relevance are presented in Table 1.

**Table 1:** Inclusion criteria for the current systematic review, including cut-offs for clinical relevance

Criteria	Description
Study design	Systematic review of RCTs. A review was considered systematic if the review authors had identified it as such.
Population	Adult (18–65 years) patients with non-specific acute LBP (onset to 6 weeks). If the systematic review contained primary studies on other populations, e.g., adolescents, at least 70% of the included studies had to be on adult populations. Findings for populations with acute LBP had to be separable from other populations.
Interventions	Interventions classified as exercise therapy (earlier defined in the background) used by physiotherapists.
Comparisons	Placebo, sham, waiting list, no treatment, usual care, minimal intervention, non-steroid anti-inflammatory drugs (NSAIDs), analgesics, or other physiotherapeutic interventions.
Outcomes	Pain intensity (hereafter referred to as pain), disability, recurrence, adverse effects.
Length of follow-up	Post-treatment, short-term (closest to three months), intermediate-term (closest to 6 months), and long-term (closest to 12 months) follow-up.
Minimal important difference (MID)^a^	15 mm on the Visual Analogue Scale (VAS) (0–100), 5 on the Roland Morris Disability Questionnaire (RMDQ) (0–24), and 10 for the Oswestry Disability Index (ODI) (0–100) [37].
Clinical relevance for pooled effect sizes	Small mean difference (MD) < 10%; medium MD 10–20%; large MD > 20% of the scale (e.g., < 10 mm on a 100 mm VAS). For relative risk: small standardized mean difference (SMD) < 0.4; medium SMD 0.41 to 0.7; large SMD > 0.7 [38].
Settings	Primary care physiotherapy or other settings in which the intervention could be practiced, such as home or gym.

*LBP* low back pain, *RCT *randomized controlled trial

^a^Based on studies presenting both anchor-based and distribution-based MID, and agreed on in consensus in an international group of experts and clinicians [37]

Excluded populations were patients with acute LBP related to pregnancy, infection, malignity, metastasis, osteoporosis, rheumatic arthritis, fracture, inflammatory process, or radiculopathy (neurologic signs).

### Search methods

#### Search strategy

We designed a comprehensive search strategy with support from a medical librarian. We took guidance from earlier published search strategies in Cochrane Reviews regarding low back pain and exercise therapy, to reach an optimal strategy. We used a wide search strategy to avoid missing relevant systematic reviews not indexed correctly in the databases. Precision of search, calculated as eligible SRs/total records, and number needed to read (NNR), calculated as 1/precision of search, were used to present the search result. No language restrictions were applied. We combined search terms and MESH terms in a search strategy developed for PubMed, and adapted this strategy for the other databases. Search strategies are presented in Additional file 2.

#### Electronic searches

We searched PubMed, Cochrane library, CINAHL, PEDro, Web of Science, Open Grey, and PROSPERO for systematic reviews from inception to 1 March 2017. The three latter were explicitly searched for grey literature, including conference abstracts and study protocols. We also searched reference lists published on the website of the McKenzie Institute International [36]. We performed a supplementary search in PubMed in September 2019. As 83% of the included systematic reviews were indexed in PubMed in the original search, we limited the update search to this database.

#### Other sources

We scrutinized the reference lists of included systematic reviews for additional potentially relevant studies. We contacted authors by email if the full text was not available.

#### Selection of systematic reviews

Two reviewers (MK and AB or SB) independently screened titles and abstracts retrieved from the searches and assessed these for eligibility against the predetermined inclusion criteria (PICOS). We retrieved all titles and abstracts meeting the inclusion criteria in full text. Two independent reviewers (MK, SB or AB) read these full text articles to assess eligibility. Disagreements between reviewers were resolved by consensus.

### Overlap

We calculated total overlap (RCTs in included reviews), and overlap for each time point, outcome, and type of exercise therapy, following the formula proposed by Pieper et al. [39]. We present overlap with percentage and corrected covered area (CCA). Interpretation of CCA: 0–5 = slight overlap, 6–10 = moderate overlap, 11–15 = high overlap, and > 15 = very high overlap.

### Assessment of methodological quality of included reviews

We used A MeaSurement Tool to Assess systematic Reviews (AMSTAR) to assess the methodological quality of the included systematic reviews [40]. AMSTAR has been shown to be a valid and reliable tool to assess methodological quality of systematic reviews [40, 41]. During the process of doing this review, an updated version, AMSTAR 2, was published [42]. Because our assessment was already in progress and the original AMSTAR has more references of reliability and validity in the literature, we chose to continue to use this tool. Two reviewers (MK, AB or SB) independently performed this assessment. Before the actual assessment, a pilot test was carried out by five reviewers (MK, SB, AB, ML, and LN) to evaluate interrater reliability for each of the eleven questions. The result showed good interrater reliability (87% agreement, Fleiss Kappa 0.58), resembling earlier tests [41]. A second pilot test was carried out by MK, AB, and SB, on another review to examine whether agreement had improved. The second test showed 100% agreement. Disagreements in the assessments were handled in a consensus dialog after comparing discrepancies between assessors.

### Data extraction

One reviewer (MK) extracted data from the included reviews and another reviewer (AB or SB) checked the extraction for accuracy. We extracted the data into a purpose-built data extraction form, adapted from a Cochrane form [43]. We extracted data primarily at the systematic review level, but supplemented this, when necessary, by extracting data at the RCT level. We verified all point estimates at the RCT level. If there were any discrepancies between an RCT and the systematic review in which it was included, we used data from the RCT. We only extracted data from populations with acute LBP. Each conclusion from the reviews and the RCTs on which this conclusion was based was extracted to enable an overall estimate of the evidence.

### Data synthesis

We synthesized the data quantitatively when possible, and otherwise qualitatively. We present the findings from the systematic reviews in summary of findings (SoF) tables for each outcome, type of exercise therapy, and time point. We present continuous data with weighted mean difference (WMD) and 95% confidence interval (CI), and dichotomous data with risk ratio (RR) and 95% CI. We used GRADEpro to create the SoF tables [44].

We considered meta-analyses feasible if clinical homogeneity in the comparisons was present, meaning that interventions, comparisons, time points, and outcomes were similar. Clinical homogeneity was assessed by at least two authors (MK and AK or SB) and agreed upon in consensus discussions. We extracted data for the meta-analyses from the original studies. To enable comparison, we rescaled the data for pain to 0–100 points (mm); e.g., a numeric pain rating score of 3 on a scale from 0 to 10 was rescaled to 30. The rescaling of the data meant that we could use WMD also for the aggregated effects, which is easier to interpret than the standardized mean difference (SMD). Effects were estimated using the inversed variance heterogeneity model, which is a robust estimation method for handling issues of underestimation of the statistical error and overconfident estimates [45]. We defined statistical significance as the 95% confidence interval not including zero. We used the free meta-analysis software MetaXL 5.3 for the statistical analyses [46].

### Assessment of certainty of evidence

To evaluate certainty in the overall body of evidence, we used the GRADE approach [47]. Certainty of evidence refers to how certain it is that the true effect of an intervention lies within a chosen range or on one side of a specified threshold [48]. In this systematic review, we used either 95% confidence intervals as the chosen range or the established minimal important difference (MID) as the specified threshold. When available, we used the GRADE and risk of bias assessments made by the authors of the included reviews, for each outcome, comparison, and time point [33]. When not available, we applied GRADE and appraised the potential limitations due to risk of bias, inconsistency, imprecision, and indirectness ourselves, based on the original studies. We did not assess publication bias due to the small number of studies in most comparisons.

## Results

### Search results

**Fig. 1:**
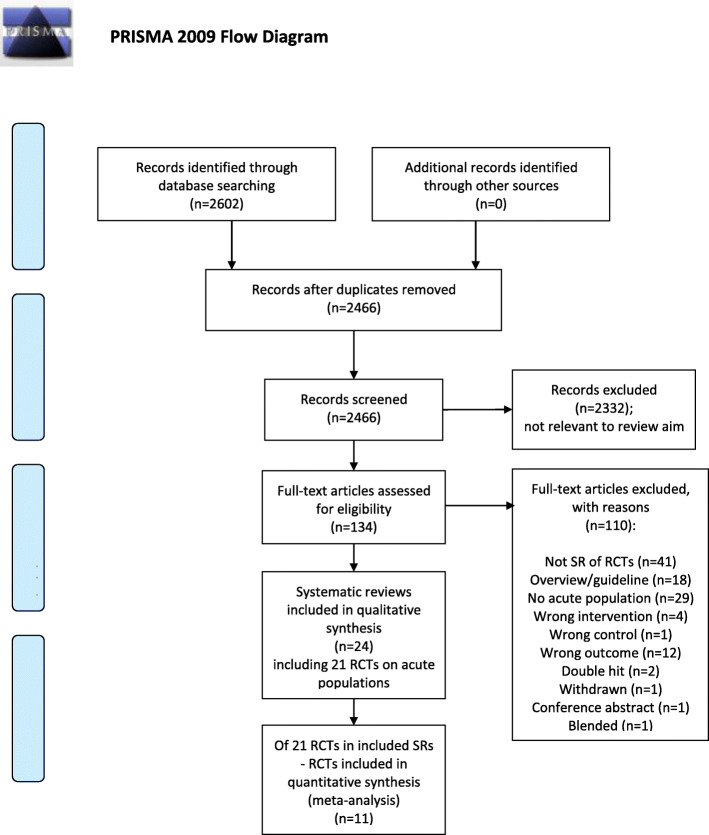
Flow diagram of the selection process

The searches retrieved 2602 records. After screening of titles and abstracts, 134 full-text assessments were carried out. We included 24 systematic reviews with a total of 572 RCTs (overlap not accounted for). Numbers needed to read was 103 and the precision of the search was 0.97%. Six of the 24 reviews were Cochrane reviews. Of the 572 RCTs, 25 publications reporting findings from 21 RCTs with a total of 2685 participants examined exercise therapy for acute LBP. Data for a total of 69 comparisons were extracted from the reviews. Eleven RCTs, with a total of 1397 participants, were included in meta-analyses. Overlap was high, 76%, with a corrected covered area of 0.14. The flowchart in Fig. 1 illustrates the selection process. The supplementary search performed in September 2019 did not result in any additional reviews that met the inclusion criteria.

### Description of included reviews

The included reviews were published between 1993 and 2018. Included RCTs in the reviews with data for an acute population were published between 1982 and 2013. Characteristics of the included reviews are presented in Table 2. Excluded reviews are presented in Additional file 3, with reason for exclusion.

**Table 2:** Characteristics of included systematic reviews and their included randomized controlled trials

First author and year in chrono-logical order	Aim of SR	Databases and search periods	No. of RCTs (of which aLBP)	Publications on RCTs (aLBP); first author, year, (no. of participants), country	Population defined in PICO of SR and described in RCT	Interventions defined in PICO of SR and described in RCT	Comparisons defined in PICO of SR and described in RCT	Outcomes defined in PICO of SR and described in RCT
Koes 1991 [49]	To determine the quality of RCTs of ET for back pain.	MEDLINE 1966–1990	16 (4)	Farrell 1982 [50] (*n* = 48) *Australia*, Waterworth 1985 [51] (*n* = 108) *New Zeeland*, Gilbert 1985/Evans 1987 [52] (*n* = 252) *Canada*, Stankovic 1990/1995 [53] (*n* = 100) *Sweden*	**Back pain.** *aLBP without neurological sign. Age: not located. Female: not located* [54]*. Mechanical aLBP. Age: not located. Female: not located* [55]*. aLBP* ± *referred pain. Age: mean 40 (SD 15). Female 49%* [51]*. aLBP* ± *referred pain. Age: 18-61. Female: 23* [56]*.*	**Physiotherapy should include individual ET provided by physiotherapists.** *Isometric flexion abdominal exercise with ergonomic instructions and microwave diathermy* [54]*. Flexion and extension exercise with ergonomic advice* [55]*.Isometric flexion exercise and education* [51]*. McK* [56]*.*	**Not defined in PICO of SR.** *Manipulation and mobilization* [54]*. NSAID with ergonomic advice or manipulation with ergonomic advice* [55]*.Bed rest, no intervention* [51]*.Mini back school* [56]*.*	**Not defined in PICO of SR.** *Time to recovery, ROM* [54]*. Pain, mobility, overall improvement, time off work, cost* [55]*. Pain, mobility, ADL* [51]*. Pain, ROM, recurrence, return to work, sick leave, patients’ ability to self-help* [56]*.*
Faas 1996 [57]	To determine from recently published trials the efficacy of exercises in patients with acute, subacute, or chronic back pain.	MEDLINE 1991 to first quarter 1995.	11 (4)	Delitto 1993 [58] (*n* = 24) *USA*, Faas 1993/1995 [59] (*n* = 473*) Netherland*,Malmivaara 1995 [60] (*n* = 186) *Finland,*Stankovic 1990/1995 [53]	**Patients with back pain.** *LBP* ± *referred pain. Age: 14–50 years. Female 42%* [61]*. LBP* ± *referred pain. Age: 16-65. Female: 43%* [62]*. LBP* ± *referred pain. Age: adults, mean 40.5. Female: 68%* [52]*. See above* [56].	**ET.** McK and mobilization [61]. Flexion exercise^68^. Extension exercise [52]. McK [56].	**Not defined in PICO of SR.** Flexion exercise [61]. Placebo ultrasound, usual care (analgesic on demand) [62]. Bed rest *for 2 days* or continue ADL *without bedrest* [52]. Mini back school [56].	**Not defined in PICO of SR.** Disability [61], pain, disability, *recurrence, ADL*, sick leave [62]. Pain, disability, *patient satisfaction, ROM, QoL, ability to work, Costs*^82^*.* Recurrence, sick leave^81^.
van Tulder 1997 [63]	To assess the effectiveness of the most common conservative types of treatment for patients with acute and chronic LBP.	MEDLINE 1966-, EMBASE 1908- and PsycLIT 1984- Sep 1995.	150 (7)	Farrell 1982 [50], Gilbert 1985/ Evans 1987 [52]Waterworth 1985 [51]*,* Stankovic 1990/1995 [53], Delitto 1993 [58], Faas 1993/1995 [59],Malmivaara 1995 [60]*.*	**Acute (0–6 weeks) or chronic LBP.** See above [51, 52, 54–56, 61, 62].	**Common conservative treatments.** See above [51, 52, 54–56, 61, 62].	**Not defined in PICO of SR.** See above [51, 52, 54–56, 61, 62].	**Pain intensity, overall improvement and functional status.** See above [51, 52, 54–56, 61, 62]*.*
van Tulder 2000 [64]	To determine whether ET is more effective than reference treatments for nonspecific LBP, and to determine which type of exercise is most effective.	MEDLINE 1966- Apr 1999. EMBASE 1988- Sep 1998.PsycLIT 1984–Apr 1999.Cochrane Library Issue 1 1999.	39 (10)	Farrell 1982 [50], Gilbert 1985/Evans 1987 [52], Waterworth 1985 [51], Stankovic 1990/1995 [53], Delitto 1993 [58], Faas 1993/1995 [59],Malmivaara 1995 [60], Cherkin 1998 [65] (*n* = 321) *Canada*, Seferlis 1998 [66] (*n* = 180) *Sweden*, Underwood 1998 [67] (*n* = 75) *United Kingdom.*	**Adults 18 to 65 years, non-specific LBP = pain located below the scapulas and above the cleft of the buttocks,** ± **radiation to the legs, including nerve root pain or sciatica.** See above [53, 63, 64, 67–70]*. LBP severe neurological sign and sciatica excluded. Age 20–64. Female 48%* [71]*. LBP* ± *sciatica requiring sick leave. Age: 19–64. Female: 47* [60]*. LBP* ± *referred pain. Age: 16–70. Female: 40%* [72]*.*	**Specific back exercises as well as abdominal, flexion, extension, static, dynamic, strengthening, stretching or aerobic exercises, if they were prescribed or performed in the treatment of LBP. Additional physicaltreatment methods were allowed.** See above [51, 52, 54–56, 61, 62]. *McK* [71]*. Intensive training program* [60]*. McK + advice* [72]*.*	**Not defined in PICO of SR.** See above [53, 63, 64, 67–70]. *Manipulation or educational booklet* [11]*.Manual therapy or General practice* [60]*.Advice with usual care* [72].	**Pain, global measure, back pain-specific functional status, return to work, ROM, generic functional status, medication use and side effects.**See above [51, 52, 54–56, 61, 62]. Global improvement, disability, cost of care, patient satisfaction, *recurrence, use of care* [71]. Pain, disability, *ROM recurrence*, patient satisfaction, days of work [60]. Pain, disability, recurrence [72].
Ferreira 2003 [73]	To assess the efficacy of manual therapy techniques in the treatment of nonspecific LBP of less than 3 months duration.	MEDLINE 1966-, EMBASE 1974-, CINAHL 1982- Mar 2001.PEDro- Jul 2002.	27 (4)	Delitto 1993 [58], Erhard 1994 [55] (*n* = 25) *USA*,Cherkin 1998 [65], Seferlis 1998 [66].	**Adults with nonspecific LBP of less than 3 months duration, as reported by the median or mean.** See above [60, 61, 71]. *LBP* ± *referred pain. Age mean 44 (SD 15). Female 38%* [50]*.*	**SMT: high-velocity, low amplitude thrust, joint manipulation; low-velocity, small- or large-amplitude joint mobilization; manual traction; or craniosacraltherapy.** See above [60, 61, 71]. McK [50].	**Not defined in PICO of SR.** See above [5, 60, 71]. *Manipulation + flexion-extension exercise* [50] ^6^*.*	**Disability, pain, QoL, adverse events, return to work, global perceived effect or patient satisfaction with therapy.** See above [60, 61, 71]. Disability [50].
Clare 2004 [70]	To investigate the efficacy of the McK method of management of non- specific spinal pain. Specific questions: What is the comparative efficacy of McK therapy in relation to inactive treatment (placebo or sham) or no treatment? What is the comparative efficacy of McK treatment in relation to other standard therapies?	MEDLINE, EMBASE, DARE, CINAHL, PEDro, CENTRAL, CDSR to Sep 2003.	6 (3)	Roberts 1990 [54] (*n* = 179) *United Kingdom*,Cherkin 1998 [65], Schenk 2003 [74] (*n* = 25) *USA*.	**Subjects with non-specific LBP or neck pain** ± **radiation. Any duration of symptoms.** See above [71]. *LBP* ± *referred pain. Age: mean 35. Female: 37%* [27]*. LBP of disc origin* ± *neurological signs. Age: 21–76. Female: 60%* [66]*.*	**Specifies individualized treatment according to the McK principles; if a co-intervention then excluded.** See above [71]. *McK* [27]. McK [66].	**No treatment, sham treatment, or another treatment.** See above [71]. *NSAID* [27]. *Mobilzation* [66].	**Pain, disability, QoL, work status, global perceived effect, medication use, medical visits, or recurrence.** See above [71]. *Pain, disability, recurrence, days of work* [27]. *Pain, disability* [66].
Hayden 2005c [69]	To assess the effectiveness of ET for reducing pain and disability in adults with non-specific acute, subacute and chronic LBP compared to no treatment, placebo, or other conservative treatments.	CENTRAL Issue 3 2004, MEDLINE, EMBASE to Oct 2004, PsychInfo, CINAHL 1999–Oct 2004.	61 (9)	Farrell 1982 [50], Gilbert 1985/Evans 1987 [52], Stankovic 1990/1995 [53], Delitto 1993 [58], Faas 1993/1995 [59], Malmivaara 1995 [60], Hides 1996/2001 [72] (*n* = 41) *Australia,* Seferlis 1998 [66], Chok 1999 [75] (*n* = 66) *Singapore*.	**Adults, acute (0–6 weeks), subacute or chronic non-specific LBP.** See above [51, 52, 54, 56, 61, 62]. *First episode of unilateral, mechanical LBP* ± *radiating pain. Age: 17–45. Female: 56%* [53]*. LBP with or without leg pain. Age: 21–54. Female: 24%* [58]*.*	**ET.** See above [51, 52, 54, 56, 61, 62].*Multifidus isometric retraining* [53]*. Endurance program* [58]*.*	**No treatment, placebo, other conservative therapy or another exercise group.** See above [51, 52, 54, 56, 61, 62]. *Advice on bed rest and absence from work with prescription of analgesic* [53]*. Non- exercise, hot pack to use at home* [58]*.*	**Pain intensity, physical functioning, global improvement and return to work/absenteeism.** See above [51, 52, 54, 56, 61, 62]. *Pain, disability, ROM, ADL, Muscle CSA* [53]*. Pain, disability, trunk extensor endurance* [58]*.*
Ferreira 2006 [68]	To conduct a SR of the effects of specific SE for spinal or pelvic pain when this intervention was compared with placebo, no treatment, another active treatment, or when specific SE was added as a supplement to other interventions.	MEDLINE 1966-, EMBASE 1974-, CINAHL 1982- and PEDro- March 2004.	12 (1)	Hides 1996/2001 [72]	**Adults with symptoms in the cervical, thoracic, low back, or pelvic area. Symptoms could be referred distal.** See above [53].	**One group received specific SE or exercise aimed at activating, training, or restoring the stabilization function of specific muscles of the spine and pelvis in isolation or in conjunction with other therapies.** See above [53].	**Not defined in PICO of SR.** See above [53].	**Disability, pain, return to work, no. of episodes, global perceived effect, or health-related quality of life.** See above [53] *+ Recurrence.*
Machado 2006 [76]	To evaluate whether the McK method is more effective than other reference treatments for acute or chronic nonspecific LBP.	MEDLINE, EMBASE, PEDro, and LILACS to Aug 2003.	11 (5)	Stankovic 1990/1995 [53], Dettori 1995 [61] (*n* = 149) *Germany*, Malmivaara 1995 [60], Cherkin 1998 [65]. Underwood 1998 [67].	**Non-specific LBP of any duration. LBP = pain between the lower rib cage and gluteal folds,** ± **radiation**. See above [52, 56, 71, 72]. *LBP* ± *referred pain. Mean age 29. Female 20%* [77]*.*	**RCTs with McK method or a synonym (McK therapy, Mechanical Diagnosis and Therapy) or intervention reflecting McK principles. Co-interventions were allowed.** See above [52, 56, 71, 72]. *McK + ice pack* [77]*.*	**Not defined in PICO of SR.** See above [52, 56, 71, 72]. *Flexion exercise or Ice pack* [20]*.*	**Pain, disability, QoL, return to work/ sick leave, or recurrence.** See above [52, 56, 71, 72]. *Pain, disability, return to work, recurrence, ROM, SLR* [77]*.*
Rackwitz 2006 [28]	To evaluate the effectiveness of segmental SE for acute, subacute and chronic LBP with regard to pain, recurrence of pain, disability and return to work.	MEDLINE 1988- and EMBASE 1989- Dec 2004.	7 (1)	Hides 1996/2001 [72].	**Adults > 18 years and take part in a program treating acute, subacute or chronic LBP** ± **sciatica.** See above [53].	**The intervention group has to have received segmental SE at least as part of the treatment.** See above [53].	**Not defined in PICO of SR.** See above [53].	**Pain, recurrence, disability, and return to work.** See above [53].
Hauggaard 2007 [78]	To evaluate the effects of specific spinal SE in patients with LBP, and to assess the methodological quality and level of evidence of the studies.	PubMed 1985- Oct 2005.PEDro 1985- Dec 2006.	10 (1)	Hides 1996/2001 [72].	**Acute, sub-acute, or chronic LBP.** See above [53].	**Intervention containing specific spinal SE including co-contraction of multifidus muscles and transversus abdominis muscles.** See above [53].	**Not defined in PICO of SR.** See above [53].	**Specific functional questionnaires and/or generic questionnaires and/or pain rating.** See above [53].
Keller 2007 [62]	To synthesize the results of RCTs for common LBP treatments comparing the interventions to placebo/ sham or no-treatment, to estimate a pooled effect size for each treatment, and compare them with each other.	CENTRAL issue 2 2005.MEDLINE, EMBASE, CINAHL, AMED from the last search in each Cochrane review to Dec 2005.	47 (4)	Faas 1993/1995 [59], Malmivaara 1995 [60], Chok 1999 [75] Mayer 2005 [79] (*n* = 100), *USA*.	**Acute and subacute/chronic LBP. Acute LBP = duration of pain less than 6 weeks.** See above [52, 58, 62]. *LBP* ± *referred pain but no neurological signs. Mean age 31.2 (SD 10.6). Female: 71%* [67]*.*	**Non-surgical treatments, including exercise, manipulation, behavioral treatment, NSAIDs, acupuncture.** See above [52, 58, 62].	**Placebo, sham treatments, no treatment, waiting list.** See above [52, 58, 62].	**Self-reported pain intensity and physical functioning.** See above [52, 58, 62].
Liddle 2007 [80]	To examine the evidence for the use of advice in management of LBP. Secondary objectives included assessment of the effectiveness of interventions in relation to LBP phase.	MEDLINE, AMED, CINAHL, PsycInfo, DARE, andCENTRAL 1985 to Sept 2004.	39 (7)	Gilbert 1985/Evans 1987 [52], Stankovic 1990/1995 [53], Faas 1993 [59], Malmivaara 1995 [60], Cherkin 1998 [65]	**Adults, 16 and 79 years with acute (0–4 weeks), subacute (4–12 weeks), or chronic (4–12 weeks) LBP.** See above [51, 52, 56, 62, 71].	**Advice, either as main intervention or as an adjunct to exercise.** See above [51, 52, 56, 62, 71].	**Placebo ultrasound, Mini back school.** See above [51, 52, 56, 62, 71].	**Back-specific function, generic health status, pain, work disability, patient satisfaction, adverse effects.** See above [51, 52, 56, 62, 71].
Engers 2008c [81]	To determine whether individual patient education is effective for pain, global improvement, functioning and return-to-work in the treatment of non-specific LBP, and to determine which type of education is most effective.	MEDLINE 1966-, EMBASE 1988-, CINAHL 1982- and PsycINFO 1984 to July 2006. CENTRAL 2006 Issue 2.	24 (2)	Cherkin 1998 [65], Mayer 2005 [79].	**Adults > 16 years with acute, subacute or chronic non-specific LBP.** See above [67, 71]	**Individual patient education.** See above [67, 71]	**No intervention, non-educational interventions or another type of individual patient education.** See above [67, 71]	**Pain intensity, global measure, back pain specific functional status, return-to-work, generic functional status, ADL.** See above [67, 71]
May 2008 [82]	To evaluate the effectiveness of SE in the treatment of pain and dysfunction from LBP.	MEDLINE 1966-, CINAHL 1982-, AMED 1985- and PEDro to Oct 2006. CENTRAL 2006 Issue 1.	18 (2)	Hides 1996/2001 [72], Brennan 2001 [83] (*n* = 123) *USA*.	**Adults > 18 years with LBP, any duration.** See above [53].*LBP* ± *referred pain in legs. No neurological signs. Age: mean 37.7 (SD 10.7). Female: 45%* [74]*.*	**One intervention arm primarily used SE = facilitation of abdominal and/or lumbar extensor muscles initially at low levels of contraction and progressing to integration into everyday activities.** See above [53]. *SE* [74]*.*	**An alternative intervention.** See above [53]. *Manipulation or DP exercises* [74]*.*	**Pain and/or functional disability.** See above [53]. *Disability* [74]*.*
Ferreira 2009 [84]	To investigate the efficacy of motor control exercises for low-back and pelvic pain.	Cochrane, MEDLINE, PEDro to 2009.	8 (1)	Hides 1996/2001 [72].	**Studies examining MCE in isolation or with other treatment.** See above [53].	**MCE according to Richardson et Jull.** See above [53].	**Not defined in SR**. See above [53].	**Pain and disability**. See above [53].
Choi 2010c [85]	To investigate the effectiveness of exercises for preventing new episodes of LBP or LBP-associated disability.	CENTRAL- 2009, issue 3, MEDLINE, EMBASE, CINAHL to July 2009.	9 (4)	Stankovic 1990/1995 [53], Faas 1993/1995 [59], Cherkin 1998 [65], Hides/1996 2001 [72].	**Adults > 18, who currently had, or had ever had at least one prior episode of non-specific LBP = defined as LBP below the costal margin and above the inferior gluteal folds** ± **leg pain, that has no specific underlying pathology.** See above [53, 56, 62, 71].	**Exercise aimed at the prevention of recurrences of LBP, divided into post-treatment and treatment. Post-treatment = exercise provided after regular treatment for an episode of back pain had been finished with the explicit aim to prevent new occurrences of back pain. Treatment = exercise for a current episode of back pain with the aim to also prevent new episodes of back pain.** See above [53, 56, 62, 71].	**Not defined in PICO of SR.** See above [53, 56, 62, 71].	**Recurrences (frequency or duration of new episodes of LBP) or the time to a LBP recurrence.** See above [53, 56, 62, 71].
Dahm 2010c [86]	To determine the effects of advice to rest in bed or stay active for patients with LBP or sciatica.	Cochrane Back Review Register to May 2009. CENTRAL 2009 issue 2. MEDLINE, EMBASE, SPORT and SCISEARCH 1998- May 2009.	10 (2)	Gilbert 1985/Evans 1987 [52], Malmivaara 1995 [60]	**Adults 16 to 80 years of age, acute (0–6 weeks) LBP=area bounded by the lowest palpable ribs superiorly and the gluteal folds inferiorly or exacerbations of chronic pain lasting less than 6 weeks.** See above [51, 52].	**One group of subjects was advised to rest in bed (instructions to stay in bed for at least two days) and at least one group was not. Or at least one group of subjects was advised to stay active (instructions to stay as active as possible and continue normal daily activities) and at least one group was not.** See above [51, 52].	**Comparison were randomized to shorter or longer periods of bed rest or to receive the advice to stay active in different ways.** See above [51, 52].	**Pain, back-specific functional status, overall disability, quality of life and adverse events.** See above [51, 52].
Kriese 2010 [71]	To evaluate the effectiveness of Segmental SE for acute, subacute, chronic and recurrent LBP.	PubMed Nov 2008–March 2009	17 (1)	Hides 1996/2001 [72].	**Acute, subacute, chronic or recurrent LBP. SR in German with abstract in English.** See above [53].	**Segmental SE.** See above [53].	**Other forms of therapy.** See above [53].	**Not defined in PICO of SR.** See above [53].
Dunsford 2011 [87]	To summarize current research evidence for DP exercises, as applied under the McK method, in the treatment of mechanical LBP.	CINAHL, AMED, MEDLINE, PubMed, EMBASE, Cochrane Library, Google Scholar, PEDro, 1995- Feb 2010.	4 (3)	Cherkin 1998 [65], Schenk 2003 [74], Mayer 2005 [79]	**Adults > 18 years, mechanical LBP of any duration with a DP.** See above [66, 67, 71].	**McK-based, DP exercises.** See above [66, 67, 71].	**All types of comparison included (either control, other conservative or surgical based intervention).** See above [66, 67, 71].	**Pain and functional outcomes were considered.** See above [66, 67, 71].
Rubinstein 2012c [23]	To examine the effectiveness of SMT for aLBP on primary and secondary outcomes as compared to inert interventions, sham, and all other treatments.	CENTRAL, MEDLINE, EMBASE,CINAHL, PEDro, and Index Chiropractic 2000 to July 2012.	20 (4)	Farrell 1982 [50], Cherkin 1998 [65], Seferlis 1998 [66], Brennan 2001 [83]	**Adults > 18 years of age with a mean duration of LBP < 6 weeks** ± **radiating pain.** See above [54, 60, 71, 74].	**Studies were included for consideration if the study design used indicated that the observed differences were due to the unique contribution of SMT.** See above [54, 60, 71, 74].	**Inert interventions, sham SMT, all other therapies or another SMT technique.** See above [54, 60, 71, 74].	**Pain, back-pain specific functional status, global improvement or perceived recovery, perceived health status or QoL and Return-to-work.** See above [54, 60, 71, 74].
Surkitt 2012 [88]	To determine the efficacy of treatment using the principles of DP Management for people with LBP and a DP.	MEDLINE 1950-, EMBASE 1980-, CENTRAL, CINAHL 1982- and PEDro to Jan 2010.	6 (2)	Schenk 2003 [74], Brennan 2001 [83]	**Trials involving male and female participants aged > 18 with LBP** ± **leg symptoms with a DP were included.** See above [66, 74].	**Trials evaluating the effect of DP management on LBP with a DP. Trials were included where DP management was used with co-interventions.** See above [66, 74].	**No therapy, placebo, or other conservative treatments.** See above [66, 74].	**Measures of pain intensity, low back-specific function, and work participation.** See above [66, 74].
Macedo 2016c [17]	To evaluate the effectiveness of motor control exercise for patients with acute non- specific LBP.	MEDLINE, EMBASE, CENTRAL, AMED to March 2015.MEDLINE In-Process and Non-Indexed Citations, CINAHL, SportDiscus, PEDro, LILACS, PubMed to April 2015.	3 (3)	Hides 1996/2001 [72], Brennan 2001 [83]Aluko 2003 [77] (*n* = 33), *United Kingdom.*	**Adults, mean age 36 (31–38). Trials with a mixed population in relation to type and duration of back pain only if separate data were provided for each group, or if the acute/subacute population corresponded to the majority of included participants (> 75%).** See above [53, 74]. LBP. Age: mean 36 (SD 9.4). Female: 85% [65].	**Trials with MCE.**See above [53, 74].*Core SE and eight specific exercises for stabilization of the transversus abdominis (TrA) and the lumbar multifidus (LM)* [65].	**No treatment, another treatment or MCE as a supplement to other interventions.** See above [53, 74]. *Core SE (specific and global trunk exercises)* [65]*.*	**Pain intensity, disability, function, quality of life, adverse events and recurrence.** See above [53, 74]. *Pain, Disability, ROM acceleration* [65]*.*
Lam 2018 [89]	To determine the effectiveness of MDT provided by trained therapists compared to that of different types of comparator interventions for improving pain and disability in patients with acute and chronic LBP separately.	MEDLINE, EMBASE, CINAHL, CDSR PsycINFO, and PEDro. Three searches: Nov, 2015, May 2016 and Sep 2017.	17 (4)	Cherkin 1998 [65], Schenk 2003 [74], Machado 2010 [27] (*n* = 146), *Australia*, Schenk 2012 [56] (*n* = 31), *USA*.	**Patients with LBP. Only trials in which therapists were MDT trained.** See above [66, 71]. aLBP, pain between the 12th rib and buttock crease, ± leg pain, < 6 weeks in duration, preceded by at least 4 weeks without LBP in which the patient did not consult a health care practitioner, 18–80 years of age. Female 50% [59]. LBP, at least 3 of 5 selection criteria from clinical prediction rules, ≥ 18 years of age mean symptom duration, 15 days. Female 61% [75].	**Studies in which an MDT classification was not completed prior to the treatment were excluded, as a priori classification is essential for the MDT approach.** See above [66, 71]. MDT: first-line care, DP exercises, postural correction and education, Treat Your Own Back book, lumbar roll, home exercise program [59]. MDT: DP exercises, home exercise program [75].	**Typical rehabilitation intervention, such as manual therapy, exercise, or education.** See above [66, 71]. Education: physician advice and acetaminophen [59]. Manual therapy plus exercise: regional lumbopelvic thrust technique, hand-heel rock range- of-motion exercise [75].	**Pain and disability.** See above [66, 71]. Pain, disability, and function [59]. Pain and disability [75].
24 SRs published from 1991 to 2018.	Sub-categories in aim: Exercise therapy in 5 SRs, conservative or common treatment in 2 SRs, comparison in 5 SRs, McK in 5 SRs and SE in 7 SRs.	19 databases/registers/Indexes included. Search range from 1908 to Sep 2017.	572 RCTs (88)^a^	25 publications based on 21 RCTs, *n* = 2685. Published from 1982 to 2013.	All RCTs include aLBP with or without referred pain in legs. Female: 47%.	Types of exercise therapy: general exercise therapy, stabilization exercise and McKenzie therapy.	34 different definitions of comparisons	22 different definitions of outcomes

Bold font = data from SR (method section). Italics = data from original RCT. c = Cochrane review

*SR* systematic review, *RCT* randomized controlled trial, *LBP* low back pain, *aLBP* acute low back pain, *ET* exercise therapy, *McK* McKenzie therapy, *SMT* spinal manipulative therapy, *NSAID* non-steroidal anti-inflammatory drug, *ROM* range of motion, *ADL* activity of daily living, *QoL* quality of life, *SE* stabilization exercise, *DP* directional preference, *MCE* motor control exercise, *MDT* mechanical diagnostic therapy, *CENTRAL* Central Register of Controlled Trials, *CDSR* Cochrane Database of Systematic Reviews

^a^Overlap not accounted for

#### Exercise therapy

Stabilization exercise and McKenzie therapy were two specific types of exercise therapy in the included reviews that were possible to classify and assess separately. When a mix of different types of exercise was used as the intervention, we used the term general exercise therapy. For stabilization exercise (including co-contraction of multifidi and transversus abdominis muscles or facilitation of abdominal and/or lumbar extensor muscles, initially at low levels of contraction with progression), various terms were used in the reviews: stabilization exercises [82], specific stabilization exercises [68], specific spinal stabilization exercises [78], segmental stabilizing exercises [28], or motor control exercises [17, 84]. McKenzie therapy [70], directional preference management [88], directional preference exercise [87], McKenzie approach [81], and McKenzie method [76, 89] were used to describe the concept officially named The McKenzie Method® of Mechanical Diagnosis and Therapy® (2018).

#### Comparisons

The following interventions were used as comparator: usual care, advice, educational booklet, general practitioner management, medical management, spinal manipulative therapy, manual therapy, NSAID, activity of daily life, no treatment, bed rest, and sham ultrasound.

#### Treatment duration and frequency

Treatment periods for the exercise therapy groups ranged from 3 days to 8 weeks. Frequency ranged from one to three visits per week. Additional home exercise frequency ranged from three times per day to once every hour.

#### Outcomes

Different outcomes were reported in the systematic reviews: pain, bothersomeness, function, functional status, disability, recurrence, patient satisfaction, global improvement, time to recovery, mobility, loss of work days, back to work, muscle thickness, sick leave, activity of daily living, and adverse effects.

#### Outcome measures

Pain was measured using the Visual Analogue Scale (VAS) or the Numeric Rating Scale (NRS). Disability was measured using the Roland Morris Disability Questionnaire (RMDQ) or the Oswestry Disability Index (ODI). Data were often transformed and presented with MD or SMD, with 95% CI or standard deviation (SD). Recurrence was measured as frequency or number of patients affected, and transformed, when possible, to risk ratio (RR) with 95% CI. Otherwise (more frequently in older systematic reviews), outcome measures from original RCTs were presented with or without a transformation to a simple negative or positive effect, with or without statistical significance (*p* value).

#### Time points

Twenty-three reviews reported post-treatment (closest to 1 week) outcomes, 19 short-term follow-up (closest to 12 weeks), two intermediate-term follow-up (closest to 26 weeks), and 20 long-term follow-up (closest to 52 weeks).

### Methodological quality of included reviews

Results of the AMSTAR quality assessment are presented in Table 3. The median overall AMSTAR score (of a maximum of 11) was 6.5 (range 2–11). Five of 24 reviews were assessed as being of high quality (AMSTAR score 9–11) [17, 23, 69, 85, 86], nine of moderate quality (AMSTAR score 5–8) [28, 64, 70, 73, 76, 80, 81, 88, 89], and ten of low quality (AMSTAR score 0–4) [49, 57, 62, 63, 68, 71, 78, 82, 84, 87]. Not reporting conflicts of interest (neither in the SRs nor in their included RCTs), followed by not reporting publication bias and a pre-determined strategy before conducting the review, were the main limitations. Reviews produced by Cochrane groups were of higher methodological quality, median 10 (range 8–11) versus non-Cochrane reviews, median 4.5 (range 2–8). Older reviews were more often of lower methodological quality; those conducted in the 1990s median 4 (range 3–4), in the 2000s median 6 (range 3–10), and in the 2010s median 8.5 (range 2–11).

**Table 3:** AMSTAR quality assessment of included reviews

AMSTAR questions												
1. Was an 'a priori' design provided?												
2. Was there duplicate study selection and data extraction?												
3. Was a comprehensive literature search performed?												
4. Was the status of publication (i.e. grey literature) used as an inclusion criterion?												
5. Was a list of studies (included and excluded) provided?												
6. Were the characteristics of the included studies provided?												
7. Was the scientific quality of the included studies assessed and documented?												
8. Was the scientific quality of the included studies used appropriately in formulating conclusions?												
9. Were the methods used to combine the findings of studies appropriate?												
10. Was the likelihood of publication bias assessed?												
11. Was the conflict of interest included?												
Review/Question	1	2	3	4	5	6	7	8	9	10	11	Sum
Koes 1991	–	–	–	–	Y	–	Y	Y	Y	–	–	4/11
Faas 1996	–	–	–	–	Y	–	Y	Y	–	–	–	3/11
vanTulder 1997	–	–	–	–	–	Y	Y	Y	Y	–	–	4/11
vanTulder 2000	Y	Y	Y	–	Y	Y	Y	Y	Y	–	–	8/11
Ferreira 2003	–	–	Y	–	Y	–	Y	Y	Y	–	–	5/11
Clare 2004	–	Y	Y	Y	Y	–	–	Y	Y	–	–	6/11
**Hayden 2005**	**Y**	**Y**	**Y**	**Y**	**Y**	**Y**	**Y**	**Y**	**Y**	**Y**	–	**10/11**
Ferreira 2006	–	–	Y	Y	–	–	–	Y	Y	–	–	4/11
Machado 2006	–	Y	Y	–	Y	Y	Y	Y	Y	–	–	7/11
Rackwitz 2006	–	Y	Y	–	Y	Y	Y	Y	Y	–	–	7/11
Hauggaard 2007	–	–	Y	–	–	Y	Y	Y	–	–	–	4/11
Keller 2007	–	–	–	–	Y	–	Y	Y	Y	–	–	4/11
Liddle 2007	–	Y	Y	Y	Y	Y	Y	Y	Y	–	–	8/11
**Engers 2008**	**Y**	**Y**	**Y**	**-**	**Y**	**Y**	**Y**	**Y**	**Y**	–	–	**8/11**
May 2008	–	–	Y	–	–	Y	–	Y	Y	–	–	4/11
Ferreira 2009	–	–	–	–	–	Y	Y	Y	–	–	–	3/11
**Choi 2010**	**Y**	**Y**	**Y**	**-**	**Y**	**Y**	**Y**	**Y**	**Y**	**Y**	–	**9/11**
**Dahm 2010**	**Y**	**Y**	**Y**	**Y**	**Y**	**Y**	**Y**	**Y**	**Y**	**Y**	–	**10/11**
Kriese 2010	–	–	–	–	–	Y	–	Y	–	–	–	2/11
Dunsford 2011	–	–	–	–	–	Y	Y	Y	Y	–	–	4/11
**Rubinstein 2012**	**Y**	**Y**	**Y**	**Y**	**Y**	**Y**	**Y**	**Y**	**Y**	**Y**	**Y**	**11/11**
Surkitt 2012	Y	Y	Y	Y	–	Y	Y	Y	Y	–	–	8/11
**Macedo 2016**	**Y**	**Y**	**Y**	**Y**	**Y**	**Y**	**Y**	**Y**	**Y**	**Y**	**Y**	**11/11**
Lam 2018	–	Y	Y	Y	–	Y	Y	Y	Y	–	–	7/11

Y = Yes; – = no or cannot answer; bold text = Cochrane review

### Quality assessment of the included RCTs, as assessed by the authors of the included reviews

Four RCTs [27, 59, 65, 77] were consistently assessed as being of high quality, two RCTs of moderate quality [54, 61], and six RCTs [50, 51, 55, 56, 58, 75] of low quality. The assessment of the remaining nine RCTs [52, 53, 60, 66, 67, 72, 74, 79, 83] varied between low and high quality. The main limitations of the RCTs were small sample sizes and lack of blinding of participants, intervention providers, and outcome assessors.

### Review conclusions for acute populations

Twenty-one of the 24 included reviews concluded that there was no difference in effects and three reviews made no conclusive statement about the difference in effect between exercise therapy and any comparator for the acute population. Three reviews concluded that there were positive effects of exercise therapy, but only for the outcome recurrence at long-term follow-up. However, this was based on the same, single RCT [72]. Of the RCTs included in the reviews, four showed results in favor of exercise therapy for some outcomes, 14 resulted in no difference, and three RCTs showed results in favor of the comparator.

### Outcomes

Findings are summarized below and presented in detail in SoF tables 4-12 (Additional file 4).

#### Pain

##### General exercise therapy

Twelve reviews [17, 23, 49, 57, 62–64, 69, 73, 76, 80, 86], including eight RCTs [50–52, 59, 61, 66, 75, 77] of low to high quality, addressed effects of general exercise therapy on pain. Overlap for the various time points ranged from 75 to 100%, with corrected covered areas of 0.25–0.70.

**Fig. 2:**
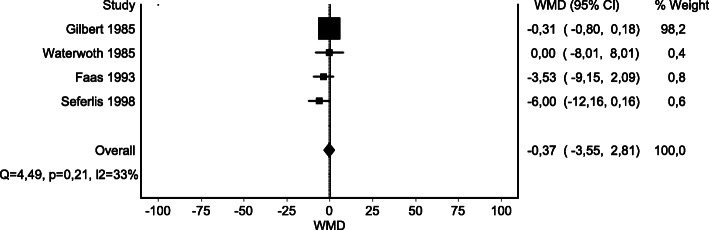
Post-treatment effects on pain of general exercise therapy versus usual care

We were able to pool data for one comparison. Meta-analysis of four RCTs [51, 59, 66, 75] comparing general exercise therapy with usual care showed no significant difference in post-treatment effects on pain (Fig. 2).

No important difference in effects of general exercise therapy on pain was reported for any comparison or time point (SoF table 4). Evidence ranging from very low to moderate certainty suggests that general exercise therapy probably results in little or no important difference in pain, at any time point, when compared with any of the investigated control interventions.

##### Stabilization exercise

Seven reviews [17, 28, 68, 71, 78, 82, 84], including three RCTs [72, 77, 83] of low to high quality, addressed effects of stabilization exercise on pain. Overlap was 67% with a corrected covered area of 0.39. No important differences in effects of stabilization exercise on post-treatment or short-term pain were reported (SoF table 5). Intermediate- or long-term effects were not reported. Evidence ranging from low to moderate certainty suggests no important difference in pain at post-treatment and short-term, when comparing stabilization exercise with other exercise therapies. The evidence is very uncertain whether stabilization exercise plus medical management reduces post-treatment pain when compared with medical management alone.

##### McKenzie therapy

Thirteen reviews [23, 49, 62–64, 69, 70, 76, 81, 86–89], including seven RCTs [27, 53, 60, 65, 67, 74, 79] of low or high quality, addressed effects of McKenzie therapy on pain. Overlap was 75% with corrected covered areas of 0.24–0.45.

**Fig. 3:**
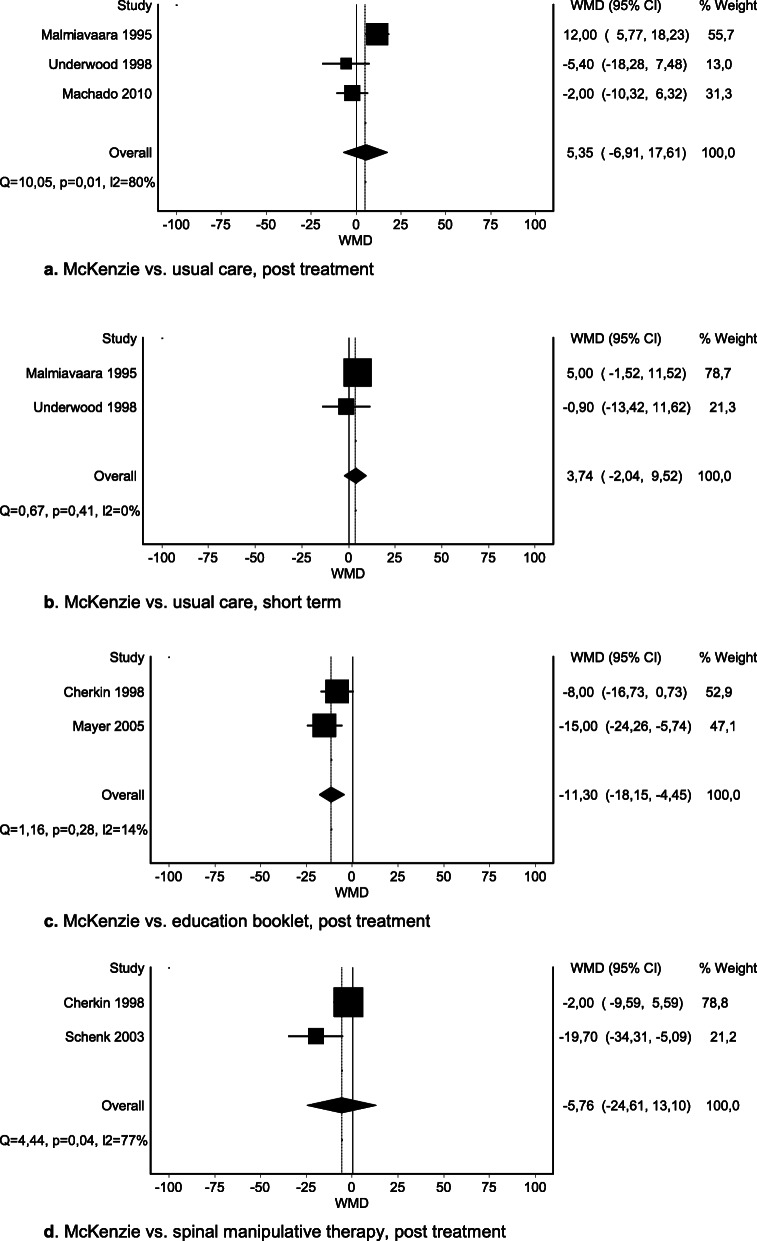
**a** McKenzie vs. usual care, post treatment. **b** McKenzie vs. usual care, short term. **c** McKenzie vs. education booklet, post treatment. **d** McKenzie vs. spinal manipulative therapy, post treatment

We were able to pool data for four comparisons. No significant difference was seen in post-treatment or short-term pain when McKenzie was compared with usual care (Fig. 3a–b) or for McKenzie therapy vs. spinal manipulative therapy post treatment (Fig. 3d). A significant difference was seen between McKenzie therapy and an educational booklet: total MD − 11.30 (95% CI − 18.15 to − 4.45) (Fig. 3c). However, the effect did not exceed the MID of 15 mm on the VAS. Findings for other comparisons are presented in SoF table 6.

At intermediate and long term, no important difference in effects of McKenzie therapy on pain was reported compared with usual care [69], educational booklet [69], spinal manipulative therapy [65], or NSAID [54]. Evidence ranging from very low to moderate certainty suggests no important difference in pain at any time point, when comparing McKenzie therapy with any of the control interventions.

#### Disability

##### General exercise therapy

Nine reviews [23, 49, 62–64, 69, 73, 80, 86], including six RCTs [50–52, 59, 66, 75] of predominantly low to moderate quality, addressed effects of general exercise therapy on disability. Overlap was 100% with corrected covered areas of 0.33–0.40.

**Fig. 4:**
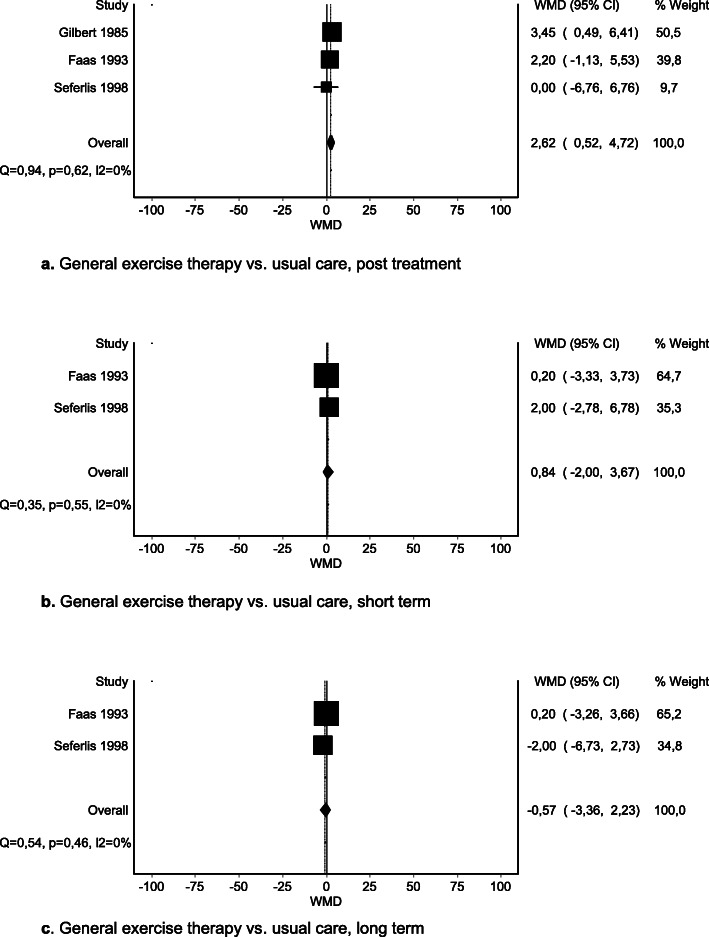
**a** General exercise therapy vs. usual care, post treatment. **b** General exercise therapy vs. usual care, short term. **c** General exercise therapy vs. usual care, long term

We were able to pool data for three comparisons. Meta-analysis of three RCTs [52, 59, 66] of general exercise therapy versus usual care showed a statistically significant difference in post-treatment effects on disability in favor of usual care: MD 2.62 (95% CI 0.52 to 4.72) (Fig. 4a). However, this effect did not exceed the MID. Meta-analysis of two RCTs [59, 66] on short-term effects and of two RCTs [59, 66] on long-term effects of disability of general exercise therapy versus usual care showed no significant difference (Fig. 4b, c, SoF table 7).

No important difference in effects of general exercise therapy on post-treatment disability was reported compared with sham ultrasound [69], spinal manipulative therapy [69], hot pack [69], or NSAID [64]. In comparison with sham ultrasound [69], hot-pack [69], bed rest, or usual care [64], no important difference in effects of general exercise therapy on short-term disability was reported. None of the included reviews reported intermediate-term effects. In comparison with sham ultrasound [69] or bed rest [64], no important difference in long-term effects of general exercise therapy on disability was reported. Evidence of low to moderate certainty suggests no important difference in disability at any time point, when comparing general exercise therapy and usual care.

##### Stabilization exercise

Seven reviews [17, 28, 68, 71, 78, 82, 84], including three RCTs [72, 77, 83] of low to high quality, addressed effects of stabilization exercise on disability. Overlap was 67% with a corrected covered area of 0.39. No important difference in effects of stabilization exercise in post-treatment, short-term, or long-term disability was reported (SoF table 8). No intermediate-term effects were reported. Evidence of very low to low certainty suggests no important difference in disability at any time point, when stabilization exercise is compared with any of the control interventions examined. The evidence is very uncertain whether stabilization exercise plus medical management reduces post-treatment disability when compared with medical management alone.

##### McKenzie therapy

Seven reviews [23, 69, 70, 76, 87–89], including seven RCTs [27, 60, 65, 67, 74, 79, 83] ranging from low to high quality, addressed effects of McKenzie therapy on disability. Overlap was 71–75% with corrected covered areas of 0.25–0.42.

**Fig. 5:**
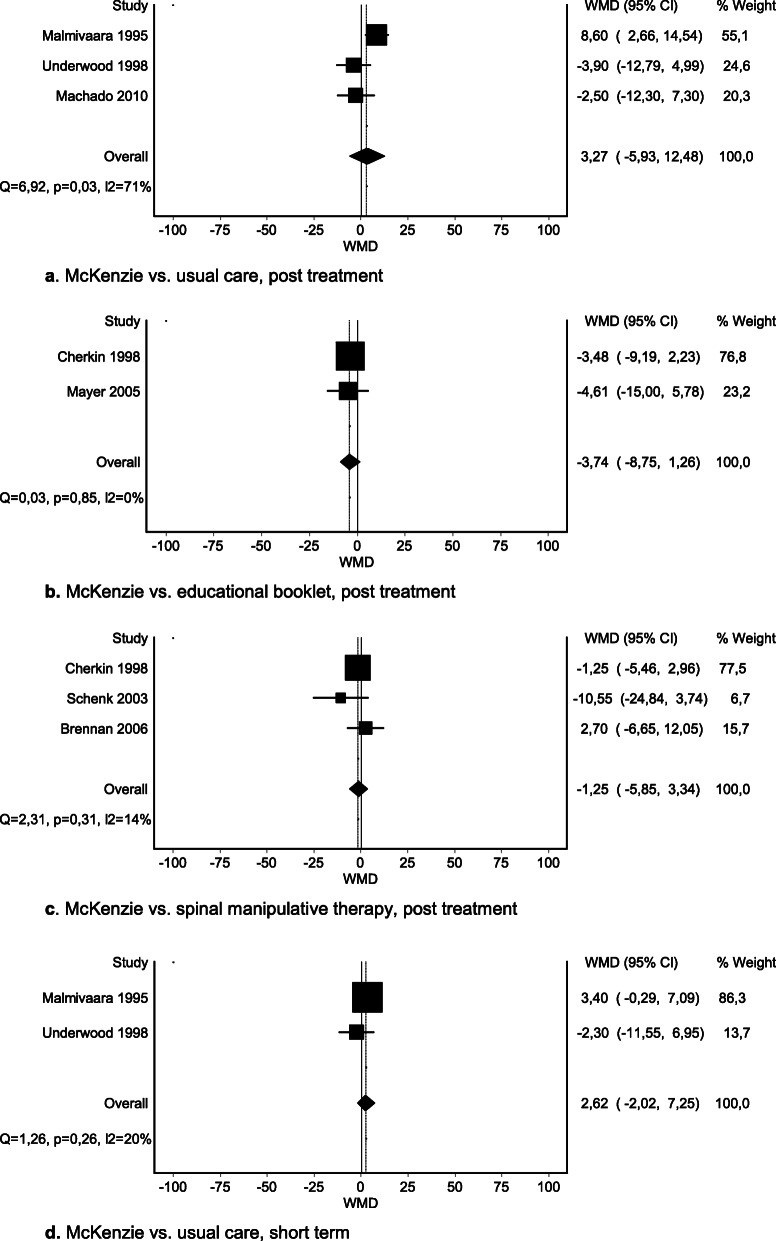
**a** McKenzie vs. usual care, post treatment. **b** McKenzie vs. educational booklet, post treatment. **c** McKenzie vs. spinal manipulative therapy, post treatment. **d** McKenzie vs. usual care, short term

We were able to pool data for four comparisons. No important differences were seen in post-treatment or short-term disability when McKenzie therapy was compared with usual care, educational booklet, or spinal manipulative therapy (Fig. 5a–d).

Findings for other comparisons are presented in SoF table 9. In comparison with educational booklet [70] or NSAID [54], no important difference in intermediate-term effects of McKenzie therapy on disability was reported. In comparison with usual care [69], educational booklet [69], spinal manipulative therapy [23], or NSAID [54], no important difference in long-term effects of McKenzie therapy on disability was reported. Evidence of very low to moderate certainty suggests that there is no difference in disability at any time point, between McKenzie therapy and usual care, spinal manipulative therapy, or NSAID. Evidence of moderate certainty suggests that McKenzie therapy likely does not reduce disability, at any time point, when compared with an educational booklet.

#### Recurrence

##### General exercise therapy

None of the included reviews addressed post-treatment, short-term, or intermediate-term effects on recurrence. Three reviews [64, 76, 85] addressed long-term effects of general exercise therapy on recurrence. Two of those [64, 85] reported results from the same RCT [59]. The third review [76] included one RCT [61] measuring long-term effects on recurrence, but this was not reported in the review. The RCTs were of moderate to high quality. Overlap was 50% with a corrected covered area of 0.25.

No significant effects of general exercise therapy on recurrence in comparison with sham ultrasound, usual care, or ice-pack was reported for any time point (SoF table 10). Evidence of moderate certainty suggests that general exercise therapy is not more effective in preventing recurrence than placebo or usual care at long-term. The evidence is very uncertain whether general exercise therapy reduces recurrence when compared with ice-pack.

##### Stabilization exercise

None of the included reviews reported post-treatment, short-term, or intermediate-term effects of stabilization exercise on recurrence. Eight reviews [17, 28, 68, 71, 78, 82, 84, 85] addressed long-term effects of stabilization exercise on recurrence, all based on one RCT [72]. Overlap was 100% with a corrected covered area of 1.0. Quality assessment of the RCT varied from low to high quality, affecting the level of evidence for stabilization exercise in the reviews, which ranged from very low to moderate evidence. Systematic reviews of low methodological quality tended to overestimate the quality of this RCT. Recurrence was measured as number of persons with recurrence in all eight reviews, and also as frequency of recurrence in one review.

Stabilization exercise plus medical management versus medical management alone resulted in lower relative risk for recurrence, RR 0.36 (95% CI 0.18 to 0.72), while there was no significant difference in recurrence frequency, MD -1.40 (95% CI -3.16 to 0.36) (SoF table 11). The evidence is very uncertain whether stabilization exercise plus medical management reduces the long-term risk for recurrence when compared with medical management alone.

##### McKenzie therapy

None of the included reviews reported any post-treatment or short-term effects of McKenzie therapy on recurrence. One review [70] addressed intermediate-term effects on recurrence but did not present results that were available in an included RCT [54], assessed as of moderate quality in the review [70]. Three reviews [70, 76, 85] addressed long-term effects on recurrence, in which three RCTs [53, 54, 65], of low to high quality, presented results for McKenzie therapy. Overlap was 50% with a corrected covered area of 0.25.

There was no difference in intermediate or long-term recurrence frequency with McKenzie therapy versus NSAID [54], and no long-term effects on recurrence compared with simple back educational interventions (SoF table 12). The evidence is very uncertain whether McKenzie therapy reduces the intermediate or long-term risk for recurrence when compared with simple back education or NSAID.

#### Adverse effects

Adverse effects were addressed in ten (42%) of the reviews [17, 23, 28, 69, 73, 80, 81, 85, 86, 88]. No review reported any adverse effects specific to the acute population. Of the included RCTs, two [54, 79] addressed adverse effects, but none of them reported any adverse effects.

## Discussion

The main findings of this systematic review are that moderate-certainty evidence suggests no superior effect of exercise therapy versus any comparator, for any of the examined outcomes, at any time point. Low-certainty evidence suggests that McKenzie therapy may be superior, with a small effect, versus simple educational booklet at post-treatment follow-up for pain; but there were no other differences between McKenzie therapy and other interventions. Very low-certainty evidence suggests that stabilization exercise together with medical management may be superior versus only medical management for recurrence at long-term follow-up, but since the evidence is of very low certainty, we cannot draw any firm conclusions; and there were no other differences between medical management and other interventions.

Adverse effects were rarely addressed in the reviews or in the included RCTs and no adverse effects were reported, indicating a possibility that they were underreported. Mild reactions with increased back pain and muscle soreness were reported in one review [69], but it was unclear in that review whether they were in the acute population or the chronic population.

The body of evidence from 24 systematic reviews consistently shows that exercise therapy in the acute phase of LBP does not yield any clinically important difference compared with any other treatment, for most outcomes and most time points. The most likely explanation for the lack of effect is the generally good prognosis (natural course) of acute LBP. The lack of long-term effect might be explained by factors such as insufficient treatment duration, frequency, or intensity of the exercise protocols. Physiology tells us that the effect of 1 to 8 weeks of exercise may not remain 1 year later, unless exercise is maintained [90]. However, relevant post-treatment and short-term effects on pain or disability are also lacking. The conclusion seems justified that the role of exercise therapy in acute LBP is very limited, at best.

The lack of effect of McKenzie therapy has been attributed in previous reviews [70, 76] to the improper use of the method, i.e., without addressing the patient’s directional preference. However, the synthesized data in our systematic review do not support any clinically relevant effect, even when this issue has been addressed. A recent review, not included in our systematic review, showed a significant difference in effects on pain and disability between RCTs that adhered to McKenzie core principles and non-adherent RCTs, but no difference between adherent RCTs and other comparators [91].

For stabilization exercise, there is no convincing benefit over other types of exercise therapy.

The most recommended “first line” care (advice to stay active and reassurance of a favorable prognosis) [12, 13] was rarely used as comparison. Instead, a wide variety of interventions, such as spinal manipulative therapy, ice-pack, hot-pack, NSAID, educational booklets, manual therapy, or medical management with prescribed bed rest, were used as comparison.

Not all included RCTs point in the direction of no effect of exercise therapy. Small RCTs tended to favor exercise therapy, suggesting a potential publication bias. When compared with spinal manipulative therapy, the results often pointed in opposite directions. In contrast, larger trials with low risk of bias pointed in the direction of no effect or no minimal important difference when exercise was compared with less strenuous interventions. The included reviews follow the same pattern; higher quality reviews report no difference, while lower quality reviews suggest a positive effect of exercise therapy. Most lower-quality reviews typically highlight a marginal effect of exercise therapy rather than stating that the effects are not clinically relevant.

The most prominent issues with regard to risk of bias and the resulting uncertainty of the evidence are the small number of RCTs in the included reviews and the small sample size of those RCTs, resulting in a lack of power to detect statistically significant differences. Lack of blinding of patients, intervention providers (which is difficult to do with these interventions), and outcome assessors were potential study limitations that further reduce our confidence in the effects of exercise therapy.

Overlap is an important issue to describe and consider when producing systematic review of systematic reviews [39]. Our systematic review showed a high overlap, which we handled by presenting it with percentage and corrected covered area for each outcome and each comparison. This minimized the risk of bias and enabled us to judge the overall certainty of evidence for the broader term of exercise therapy and the two more specific types, i.e., stabilization exercise and McKenzie therapy.

We are not aware of any other systematic review of systematic reviews addressing exercise therapy for acute LBP. Swinkels et al. [92] addressed the effect of exercise therapy for nonspecific LBP in their overview. That overview included four reviews of which two [57, 69] were included in our systematic review. The other two reviews addressed non-acute populations. Swinkels et al. [92] concluded, based on the study by Hayden et al. [69], that exercise therapy is as effective as either no treatment or other non-exercise interventions at short-, intermediate-, and long term follow-up. We do not disagree with that conclusion but conclude, based on the studies included in our systematic review, that exercise therapy does not result in any minimal important difference in effect compared with other interventions. Maher et al. [93] concluded, also based on the study by Hayden et al. [69], that high-quality evidence exists for no difference between exercise therapy versus sham treatment or other conservative treatments. While we agree with their conclusion, our analysis only supports moderate certainty of evidence for this comparison, suggesting that future studies may change our confidence in the estimate of effects.

An updated publication of recommendations in international clinical guidelines showed that exercise therapy is recommended for acute LBP in three of 14 guidelines and that the other 11 guidelines provided inconsistent recommendations on exercise therapy for acute LBP [94]. The Danish guidelines [95] recommend exercise therapy based on low-quality evidence from seven RCTs, including a population with acute LBP (in their definition up to 12 weeks’ duration). The authors made the recommendation based on a trend in the results favoring exercise therapy. Five of these RCTs are included in our systematic review. Our findings do not support these recommendations. However, recommendations in guidelines are based on more aspects than solely evidence from systematic reviews. Patients’ preferences, clinicians’ experiences, costs, availability, and safety are examples of other aspects that are considered and which could explain the discrepancy between recommendations and evidence.

Some of the limitations in this systematic review may have introduced potential biases. The low methodological quality of some of the included reviews and their underlying RCTs contributes to the low certainty of evidence. For most outcomes and time points, the total number of participants was low. The inclusion of the same RCTs in many of the reviews caused a high overlap, and the varying results of review authors’ methodological quality assessment of some RCTs is a further cause for concern. Furthermore, our meta-analyses were based on aggregate data, which entails a potential for ecological fallacy. A difference in outcomes can be significant in several subgroups, but when combined, this difference may disappear or even reverse; a fallacy known as Simpson’s paradox [96]. Our overall GRADE assessment was based on a combination of assessments made by the systematic review authors and ourselves. This combination may entail inconsistency in assessments, as reliability between the assessment made by the authors of the systematic reviews and our research group is unknown.

We have followed available methodological guidance for conducting a systematic review of systematic reviews, but the guidance is evolving and several strategies are available. The choice of strategy will have an impact on the results and conclusion. Overlap could have been minimized by excluding reviews with the same RCTs included. Another possible strategy would have been to exclude reviews of lower methodological quality. However, then we would not have obtained a complete picture of the overall certainty of the evidence from all available reviews.

### Implications for practice

LBP is among the most common reasons for which patients consult a physiotherapist or general practitioner in primary care [97]. It is important to provide accurate, timely, and effective management for this condition. The findings of this systematic review of systematic reviews do not suggest any benefit of using exercise therapy in the acute phase of LBP. None of the exercise types resulted in any effects, in any of the comparisons, which exceeded the established minimal important difference for pain and function [37]. This was true both when compared with placebo (sham ultrasound), with less strenuous interventions (advice to stay active, reassurance of an optimistic prognosis, and educational booklet), and with other forms of exercise therapy. This is important knowledge that the physiotherapist and general practitioner need to adopt in their clinical practice. Exercise is still used by physiotherapists for acute LBP, although not to as great extent as for subacute or chronic LBP [9]. Our findings imply that physiotherapists and general practitioners should be more reluctant in providing exercise therapy for acute LBP, and instead more strongly stress the good prognosis and provide reassurance and advice to stay active. They also need to communicate this knowledge to their patients with acute LBP so that patient and therapist can make an informed treatment decision together. Good patient–therapist communication is essential to achieve a collaborative rehabilitation and engage patients in their treatment [98]. In accordance with the principles of evidence-based practice, the physiotherapist and general practitioner should integrate their patient’s preferences and values with their own clinical expertise and the research findings, to determine if and when exercise therapy could, or should, be the intervention of choice.

### Future research

This systematic review of systematic reviews reveals many areas in which there is room for improvement in terms of rigorous conduct of RCTs that would enhance certainty of the evidence. Such improvement is necessary if we are to come to a more certain conclusion regarding the effects or non-effects of exercise therapy for acute LBP. Increasing sample size to reach sufficient power, standardizing outcomes and outcome measures, choosing relevant time points and relevant comparisons, and improving the reporting of conflicts of interest are some issues that would strengthen the certainty of evidence. Presenting study findings with minimally important differences and confidence intervals would enhance applicability of the findings.

Assigning more weight to results from studies with low risk of bias or excluding studies with high risk of bias are strategies that could resolve discrepancies in existing reviews and their included RCTs. Our systematic review found more systematic reviews than RCTs. In view of the many systematic reviews published and the large extent of overlap, the need to conduct another systematic review is limited, unless new RCTs are published. However, it is questionable whether it would be worthwhile to invest public funding in new trials on exercise therapy for acute LBP. Small trials with a high risk of bias are expected to overestimate the true effect. If 24 systematic reviews and 21 RCTs do not show a clinically important effect of exercise therapy for acute LBP, scarce resources for research might be better spent on prevention or treatment of chronic LBP. Reducing the enormous burden of chronic LBP seems a priority world-wide [99].

We found the GRADE approach challenging to apply and believe it would benefit from further development and guidance for use in systematic reviews of systematic reviews, to facilitate assessment of the certainty of the evidence. GRADE was developed to assess certainty of evidence based on risk of bias and other criteria in primary studies, whereas in a systematic review of systematic reviews, the unit of analysis is the included reviews. Methodological quality of the underlying RCTs is an important component of the GRADE assessment, and we had to rely on satisfactory quality appraisal and reporting by the review authors. However, we found considerable variation in the quality appraisal, and hence GRADE assessment, among the included reviews. We attempted to use Pollocks et al.’s algorithm for assigning GRADE levels [100], but did not find it suitable for this systematic review of systematic reviews. Greater consistency is needed with regard to how systematic review authors extract and present data and assess evidence, so that systematic review authors can rely on the underpinning data without having to go back to the original RCTs. Using GRADE for each outcome, time point, and intervention is feasible as long as the overlap is controlled in systematic reviews of systematic reviews.

### Ethical considerations

When more systematic reviews than RCTs are conducted in a certain field, we need to consider other approaches to get answers. Doing more underpowered or biased RCTs is likely to further increase inconsistency and heterogeneity. Comparing one intervention to another, where none of the interventions have any superior effect compared with no treatment or sham, will not increase the certainty of evidence. Maybe the right question to ask is why large RCTs are still missing, despite three decades of systematic reviews based on RCTs? Faas et al. (74) studied 493 participants in 1993 and since then, no other RCT has succeeded in matching this number of participants. Why? Patients are often the ones who participate in the underpowered and biased studies and in the end the ones who receive the interventions. It does not seem appropriate or ethical to continue including patients in trials that will not adequately answer the research question.

## Conclusions

The findings of this systematic review of systematic reviews suggest that there is very low-to-moderate certainty evidence that exercise therapy of any type may result in little or no important difference in pain or disability in adult patients with acute LBP, compared with other interventions, at any of the follow-up points reported. It is uncertain whether stabilization exercise in the acute phase reduces the risk of recurrence. Contradictory findings were seen in some small RCTs of low methodological quality. Adverse effects seem rare, but the total sample is too small to draw firm conclusions.

Knowledge about the certainty of evidence for the effectiveness of exercise therapy is important for the physiotherapist in clinical primary care practice and should be used to inform treatment decisions. The knowledge should be communicated to the patient, together with other treatment options, so that a fully informed, joint decision about treatment can be made.

## Supplementary information

**Additional file 1.** PRISMA checklist.

**Additional file 2.** Search strategies.

**Additional file 3.** Excluded systematic reviews.

**Additional file 4.** Summary of Findings Tables 4-12.

## Data Availability

All data supporting the findings of this systematic review are included in this published article and its supplementary files.

## Abbreviations

Acute LBP: Acute low back pain
AMSTAR: A MeaSurement Tool to Assess systematic Reviews
CCA: Corrected covered area
MID: Minimal important difference
NRS: Numeric Rating Scale
NSAID: Non-steroid anti-inflammatory drug
ODI: Oswestry Disability Index
PICOS: Patient, Intervention, Comparison/Control, Outcome, Setting/Study design
PROSPERO: International prospective register of systematic reviews
PRISMA: Preferred Reporting Items for Systematic Review and Meta-Analysis Protocols
RMDQ: Roland Morris Disability Index
SoF: Summary of findings
VAS: Visual Analogue Scale
