# Supplementary material for: Effects of exercise therapy in patients with acute low back pain: a systematic review of systematic reviews
Source: Syst Rev. 2020 Aug 14;9:182. doi: 10.1186/s13643-020-01412-8 (PMC7427286; doi:10.1186/s13643-020-01412-8)
Supplement: Supplementary file 2 — Additional file 2. Search strategies. [file 13643_2020_1412_MOESM2_ESM.docx]

# Additional file 2. Search strategies

PubMed

1. low back pain.mesh
2. back pain.mesh
3. “low back pain”.ti,ab
4. “back pain”.ti,ab
5. backache.ti,ab
6. “back ache”.ti,ab
7. “back strain”. ti,ab
8. lumbago. ti,ab
9. “lumbar pain”. ti,ab
10. “lumbar disorder”. ti,ab
11. “lumbar disorders”.ti,ab
12. “lumbar dysfunction”. ti,ab
13. “lumbar dysfunctions”. ti,ab
14. dorsalgia.ti,ab.
15. “spinal pain”.ti,ab
16. “spinal disorder”.ti,ab
17. “spinal disorders”.ti,ab
18. “spinal dysfunction”.ti,ab
19. “spinal dysfunctions”.ti,ab
20. “postural pain”.ti,ab
21. spine.ti.ab
22. or/1-21
23. exercise.mesh.
24. exercise therapy.mesh.
25. exercise movement techniques.mesh.
26. “exercise therapy”.ti,ab.
27. “exercise therapies”. ti,ab.
28. exercise.ti,ab.
29. exercises.ti,ab.
30. physical therapy modalities.mesh.
31. “motor control exercise”. ti,ab.
32. “motor control exercises”. ti,ab.
33. “stabilisation exercise”. ti,ab.
34. “stabilisation exercises”. ti,ab.
35. “stabilization exercise”. ti,ab.
36. “stabilization exercises”. ti,ab.
37. “stabilisation training”. ti,ab.
38. “stabilization training”. ti,ab.
39. “stability exercise”. ti,ab.
40. “stability exercises”. ti,ab.
41. “stability training”. ti,ab.
42. “core exercise”. ti,ab.
43. “core exercises”. ti,ab.
44. “core training”. ti,ab.
45. “core stability”. ti,ab.
46. mckenzie. ti,ab.
47. “mechanical diagnosis and therapy”. ti,ab.
48. hydrotherapy. ti,ab.
49. hydrotherapies. ti,ab.
50. balneotherapy. ti,ab.
51. balneotherapies. ti,ab.
52. “balnear therapy”. ti,ab.
53. “balnear therapies”. ti,ab.
54. “segmental exercise”. ti,ab.
55. “segmental exercises”. ti,ab.
56. “segmental training”. ti,ab.
57. walking. ti,ab.
58. walk. ti,ab.
59. physiotherapy. ti,ab.
60. physiotherapies. ti,ab.
61. “physical therapy”. ti,ab.
62. “physical therapies”. ti,ab.
63. “physical activity“.ti,ab.
64. “physical activities“.ti,ab.
65. or/23-64
66. systematic review.pt
67. meta analysis.pt
68. review.pt
69. “systematic review”.ti,ab.
70. “meta analysis”. ti,ab.
71. overview. ti,ab.
72. or/66-71
73. humans.fi
74. **22 and 65 and 72 and 73**

The Cochrane Library

1. "low back pain".ti,ab,kw.

2. "back pain".ti,ab,kw.

3. lumbago.ti,ab,kw.

4. dorsalgia.ti,ab,kw.

5. "spinal pain".ti,ab,kw.

6. "spinal disorder".ti,ab,kw.

7. "spinal dysfunction".ti,ab,kw.

8. "lumbar pain".ti,ab,kw.

9. "lumbar disorder".ti,ab,kw.

10. "lumbar dysfunction".ti,ab,kw.

11. "postural pain".ti,ab,kw.

12. backache.ti,ab,kw.

13. "back ache".ti,ab,kw.

14. “back strain”.ti,ab,kw.

15. spine.ti,ab,kw.

16. low back pain.mesh(explode all trees).

17. back pain.mesh(explode all trees).

**18. or/1-17**

19. exercise.ti,ab,kw.

20. "exercise therapy”.ti,ab.kw.

21. "exercise therapy modalities”.ti,ab,kw.

22. "motor control exercise”.ti,ab,kw.

23. "stabilization exercise".ti,ab,kw.

24. "stabilisation exercise”.ti,ab,kw.

25. "stability exercise”.ti,ab,kw.

26. "stability training".ti,ab,kw.

27. “core exercise".ti,ab,kw.

28. "core training".ti,ab,kw.

29. "core stability".ti,ab,kw.

30. "segmental exercise".ti,ab,kw.

31. “conservative treatment”.ti,ab,kw.

32. “conservative intervention”.ti,ab,kw.

33. “physical therapy”.ti,ab,kw.

34. physiotherapy.ti,ab,kw.

35. hydrotherapy.ti,ab,kw.

36. balneotherapy.ti,ab,kw.

37. “physical activity”.ti,ab,kw.

38. “segmental training”.ti,ab,kw.

39. mckenzie.ti,ab,kw.

40. “directional preference”.ti,ab,kw.

41. “mechanical diagnosis and therapy”.ti,ab,kw.

42. walk.ti,ab,kw.

43. walking.ti,ab,kw.

**44. or 19-43**

**45. 19 and 44**

CINAHL

1. "low back pain".ab.

2. "back pain".ab.

3. lumbago.ab.

4. dorsalgia.ab.

5. "spinal pain".ab.

6. "spinal disorder".ab.

7. "spinal dysfunction".ab.

8. "lumbar pain".ab.

9. "lumbar disorder".ab.

10. "lumbar dysfunction".ab.

11. "postural pain".ab.

12. backache.ab.

13. "back ache".ab.

14. “back strain”.ab.

15. spine.ab.

**16. or/1-15**

17. exercise.ab.

18. "exercise therapy”.ab.

19. "exercise therapy modalities”.ab.

20. "motor control exercise”.ab.

21. "stabilization exercise".ab.

22. "stabilisation exercise”.ab.

23. "stability exercise”.ab.

24. "stability training".ab.

25. “core exercise".ab.

26. "core training".ab.

27. "core stability".ab.

28. "segmental exercise".ab.

29. “conservative treatment”.ab.

30. “conservative intervention”.ab.

31. “physical therapy”.ab.

32. physiotherapy.ab.

33. hydrotherapy.ab.

34. balneotherapy.ab.

35. “physical activity”.ab.

36. “segmental training”.ab.

37. mckenzie.ab.

38. “directional preference”.ab.

39. “mechanical diagnosis and therapy”.ab.

40. walk.ab.

41. walking.ab.

**42. or 17-41**

**45. 17 and 42**

-Exclude MEDLINE-poster

-Publication type: Systematic Review, Meta-Analysis or Review

-Human filter

-Adapt related words

-Find all search terms

PEDro

Abstract and title: back

Problem: pain

Body part: lumbar spine, sacro- iliac joint or pelvis

Method: systematic review

When searching: match all search terms (AND)

Web of Science

You searched for: TITLE: ("back pain")

Refined by:  DOCUMENT TYPES: (MEETING ABSTRACT OR REVIEW ) AND  DOCUMENT TYPES: ( MEETING ABSTRACT ) AND  TOPIC:("systematic review")

Timespan: All years.  Indexes: SCI-EXPANDED, SSCI, A&HCI, CPCI-S, CPCI-SSH, ESCI.

Open Grey

**Title** “back pain” and “systematic review”

Prospero

Review Title: Exercise therapy for acute low back pain: a systematic review of systematic reviews

Review Question: The aim of this systematic review of systematic reviews will be to assess the overall certainty in the evidence for the effect of exercise therapy used for patients with acute low back pain.

What are the effects of exercise therapy on pain intensity?

What are the effects of exercise therapy on functional status/ disability?

What are the effects of exercise therapy on recurrence?

What are the effects of exercise therapy on patient satisfaction?

What are the effects of exercise therapy on global improvement or perceived recovery?

What are the reported adverse effects in the systematic reviews?

Condition/Domain: Acute episode of low back pain. Primary care setting. Physical therapy.

Participants/Population: Population: Adult (18-65 years) patients with non-specific acute (from onset up to six weeks) low back pain.

Comparator: Placebo/sham, wait list, no treatment, usual practice, minimal intervention, NSAID, analgesic or other physical therapy interventions.

Outcome: Primary: pain intensity measured with visual analogue scale (VAS 0-100) or a numerical rating scale (NRS 0-10). Functional status/ disability measured with Roland Disability Questionnaire or the Oswestry Disability Index. Recurrence measured as relative risk or risk ratio. Secondary: Patient satisfaction, global improvement or perceived recovery, and adverse effects.
