# Supplementary material for: Effects of exercise therapy in patients with acute low back pain: a systematic review of systematic reviews
Source: Syst Rev. 2020 Aug 14;9:182. doi: 10.1186/s13643-020-01412-8 (PMC7427286; doi:10.1186/s13643-020-01412-8)
Supplement: Supplementary file 3 — Additional file 3. Excluded systematic reviews. [file 13643_2020_1412_MOESM3_ESM.docx]

# Additional file 3. Excluded systematic reviews in order of publication, with reason for exclusion

| **No.** | **Record** | **Reason for exclusion** |
| --- | --- | --- |
| 1. | Asenlof P, Michaelsson P, Grahn B, Bergman S, Axelsson S, Gyllensward H, et al. A systematic review of randomised controlled trials studying the preventive effects of physical exercise, manual and behavioural treatments in acute low back pain and neck pain. Int J Behav Med. 2016;23:S187-S. | Conference abstract |
| 2. | Davin J, Callaghan M. Towards evidence based emergency medicine: Best BETs from the Manchester Royal Infirmary. BET 2: Core stability versus conventional exercise for treating non-specific low back pain. Emerg Med J. 2016;33(2):162-3. | Not SR |
| 3. | Gomes-Neto M, Lopes JM, Conceicao CS, Araujo A, Brasileiro A, Sousa C, et al. Stabilization exercise compared to general exercises or manual therapy for the management of low back pain: a systematic review and meta-analysis [with consumer summary]. Phys Ther Sport. 2016 Aug 18:Epub ahead of print. 2016. | Wrong P, chronic LBP |
| 4. | Goode AP, Coeytaux RR, McDuffie J, Duan-Porter W, Sharma P, Mennella H, et al. An evidence map of yoga for low back pain. Complement Ther Med. 2016;25:170-7. | Wrong P, chronic LBP |
| 5. | Ishak NA, Zahari Z, Justine M. Effectiveness of strengthening exercises for the elderly with low back pain to improve symptoms and functions: a systematic review. Scientifica. 2016;(3230427):Epub. 2016. | Wrong P, chronic LBP |
| 6. | Nicolson PJA, Bennell KL, Dobson FL, Van Grackel A, Holden MA, Hinman RS. Interventions to increase adherence to therapeutic exercise in older adults with low back pain and/or osteoarthritis: a systematic review and meta- analysis. Int J Behav Med. 2016;23:S259-S. | Wrong O, adherence. |
| 7. | Patrick N, Emanski E, Knaub MA. Acute and chronic low back pain. Med Clin North Am. 2016;100(1):169-81. | Not SR |
| 8. | Steffens D, Maher CG, Pereira LS, Stevens ML, Oliveira VC, Chapple M, et al. Prevention of Low Back Pain: A systematic review and meta-analysis. JAMA Intern Med. 2016;176(2):199-208. | Wrong O and P |
| 9. | Yamato TP, Maher CG, Saragiotto BT, Hancock MJ, Ostelo R, Cabral CMN, et al. Pilates for low back pain: complete republication of a Cochrane review [with consumer summary]. Spine. 2016 Jun;41(12):1013-1021. 2016. | Wrong P, chronic LBP |
| 10. | Schaafsma FG, Anema JR, van der Beek AJ. Back pain: Prevention and management in the workplace. Best Pract Res Clin Rheumatol. 2015;29(3):483-94. | Not SR |
| 11. | Saragiotto BT, Yamato TP, Maher C. Yoga for low back pain: PEDro systematic review update. Br J Sports Med. 2015;49(20):1351. | Not SR |
| 12. | Mazzarino M, Kerr D, Wajswelner H, Morris ME. Pilates method for women's health: Systematic review of randomized controlled trials. Arch Phys Med Rehabil. 2015;96(12):2231-42. | Wrong P, chronic LBP |
| 13. | Hidalgo B, Detrembleur C, Hall T, Mahaudens P, Nielens H. The efficacy of manual therapy and exercise for different stages of non-specific low back pain: an update of systematic reviews. J Man Manip Ther. 2014 May;22(2):59-74. 2014. | Wrong I |
| 14. | Menke JM. Do manual therapies help low back pain? A comparative effectiveness meta-analysis. Spine (Phila Pa 1976). 2014;39(7):E463-72. | Wrong I |
| 15. | Smith BE, Littlewood C, May S. An update of stabilisation exercises for low back pain: a systematic review with meta-analysis. BMC Musculoskelet Disord. 2014;15:416. | Wrong P, chronic LBP |
| 16. | Wong AYL, Parent EC, Funabashi M, Kawchuk GN. Do changes in transversus abdominis and lumbar multifidus during conservative treatment explain changes in clinical outcomes related to nonspecific low back pain? A systematic review [with consumer summary]. J Pain. 2014 Apr;15(4):377e371-377e335. 2014. | Wrong I |
| 17. | Becker JA, Stumbo JR. Back pain in adults. Prim Care. 2013;40(2):271-88. | Not SR |
| 18. | Brumitt J, Matheson JW, Meira EP. Core stabilization exercise prescription, part 2: a systematic review of motor control and general (global) exercise rehabilitation approaches for patients with low back pain. Sports Health 2013. Nov-Dec;5(6):510-513. 2013. | Wrong P, chronic LBP |
| 19. | Bystrom MG, Rasmussen-Barr E, Grooten WJ. Motor control exercises reduces pain and disability in chronic and recurrent low back pain: a meta-analysis. Spine (Phila Pa 1976). 2013;38(6):E350-8. | Wrong P, chronic LBP |
| 20. | Macedo LG, Bostick GP, Maher CG. Exercise for prevention of recurrences of nonspecific low back pain. Phys Ther. 2013;93(12):1587-91. | Not SR |
| 21. | Richards MC, Ford JJ, Slater SL, Hahne AJ, Surkitt LD, Davidson M, et al. The effectiveness of physiotherapy functional restoration for post-acute low back pain: a systematic review. Man Ther. 2013;18(1):4-25. | Wrong I |
| 22. | Sharma M, Haider T. Yoga as an alternative and complementary treatment for patients with low back pain: a systematic review. J Evid Based Complementary Altern Med. 2013 Jan;18(1):23-28. 2013. | Wrong P, chronic LBP |
| 23. | Ward L, Stebbings S, Cherkin D, Baxter GD. Yoga for functional ability, pain and psychosocial outcomes in musculoskeletal conditions: a systematic review and meta-analysis. Musculoskelet Care. 2013;11(4):203-17. | Wrong P, chronic LBP |
| 24. | Schaafsma FG, Whelan K, van der Beek AJ, van der Es-Lambeek LC, Ojajarvi A, Verbeek JH. Physical conditioning as part of a return to work strategy to reduce sickness absence for workers with back pain. Cochrane Database Syst Rev. 2013(8):Cd001822. | Wrong I and O |
| 25. | Bussing A, Ostermann T, Ludtke R, Michalsen A. Effects of yoga interventions on pain and pain-associated disability: a meta-analysis. J Pain. 2012;13(1):1-9. | Not SR |
| 26. | Casazza BA. Diagnosis and treatment of acute low back pain. Am Fam Physician. 2012;85(4):343-50. | Not SR |
| 27. | Guild DG. Mechanical therapy for low back pain. Prim Care. 2012;39(3):511-6. | Not SR |
| 28. | Kuczynski JJ, Schwieterman B, Columber K, Knupp D, Shaub L, Cook CE. Effectiveness of physical therapist administered spinal manipulation for the treatment of low back pain: a systematic review of the literature. Int J Sports Phys Ther. 2012 Dec;7(6):647-662. 2012. | Wrong I and P |
| 29. | Laird RA, Kent P, Keating JL. Modifying patterns of movement in people with low back pain -does it help? A systematic review. BMC Musculoskelet Disord. 2012;13:169. | Wrong I |
| 30. | Lizier DT, Perez MV, Sakata RK. Exercises for treatment of nonspecific low back pain. Rev Bras Anestesiol. 2012;62(6):838-46. | Not SR |
| 31. | Oliveira VC, Ferreira PH, Maher CG, Pinto RZ, Refshauge KM, Ferreira ML. Effectiveness of self-management of low back pain: systematic review with meta-analysis [with consumer summary]. Arthritis Care Res. 2012 Nov;64(11):1739-1748. 2012. | Wrong I |
| 32. | Posadzki P. Is spinal manipulation effective for pain? An overview of systematic reviews. Pain Med. 2012;13(6):754-61. | Wrong P |
| 34. | Verhagen AP, Cardoso JR, Bierma-Zeinstra SM. Aquatic exercise & balneotherapy in musculoskeletal conditions. Best Pract Res Clin Rheumatol. 2012;26(3):335-43. | Not SR |
| 35. | Carter C, Stratton C, Mallory D. Yoga to treat nonspecific low back pain. AAOHN J. 2011;59(8):355-61; quiz 62. | Not SR |
| 36. | McIntosh G, Hall H. Low back pain (acute). BMJ Clin Evid. 2011;2011. | Not SR |
| 37. | Muller R, Linz W, Buchmann J. [Concept of treatment of low back pain]. MMW Fortschr Med. 2011;153(1-2):31-3. | Not SR |
| 38. | Posadzki P, Ernst E. Spinal manipulation: an update of a systematic review of systematic reviews. N Z Med J. 2011;124(1340):55-71. | Not SR |
| 39. | Posadzki P, Ernst E. Yoga for low back pain: a systematic review of randomized clinical trials. Clin Rheumatol. 2011;30(9):1257-62. | Wrong P, chronic LBP |
| 40. | Posadzki P, Lizis P, Hagner-Derengowska M. Pilates for low back pain: a systematic review. Complement Ther Clin Pract. 2011;17(2):85-9. | Wrong P, chronic LBP |
| 41. | The Lumbosacral Spine: Kinesiology, physical rehabilitation, and interventional pain medicine. Clin Kinesiol (Online Edition). 2010:22-50. | Not SR |
| 42. | Artus M, van der Windt DA, Jordan KP, Hay EM. Low back pain symptoms show a similar pattern of improvement following a wide range of primary care treatments: a systematic review of randomized clinical trials [with consumer summary]. Rheumatology. 2010 Dec;49(12):2346-2356. 2010. | Wrong O |
| 43. | Behm DG, Drinkwater EJ, Willardson JM, Cowley PM. The use of instability to train the core musculature. Appl Physiol Nutr Metab. 2010;35(1):91-108. | Not SR |
| 44. | Hendrick P, Te Wake AM, Tikkisetty AS, Wulff L, Yap C, Milosavljevic S. The effectiveness of walking as an intervention for low back pain: a systematic review. Eur Spine J. 2010;19(10):1613-20. | Wrong P, chronic LBP |
| 45. | Kent P, Mjosund HL, Petersen DH. Does targeting manual therapy and/or exercise improve patient outcomes in nonspecific low back pain? A systematic review. BMC Med. 2010;8:22. | Wrong O |
| 46. | Bell JA, Burnett A. Exercise for the primary, secondary and tertiary prevention of low back pain in the workplace: a systematic review. J Occup Rehabil. 2009;19(1):8-24. | Wrong O |
| 47. | Bigos SJ, Holland J, Holland C, Webster JS, Battie M, Malmgren JA. High-quality controlled trials on preventing episodes of back problems: systematic literature review in working-age adults. Spine J. 2009;9(2):147-68. | Wrong O |
| 48. | Chou R, Atlas SJ, Stanos SP, Rosenquist RW. Nonsurgical interventional therapies for low back pain: a review of the evidence for an American Pain Society clinical practice guideline. Spine (Phila Pa 1976). 2009;34(10):1078-93. | Wrong I |
| 49. | Lee MS, Pittler MH, Ernst E. Internal qigong for pain conditions: a systematic review. J Pain. 2009;10(11):1121-7.e14. | Wrong P, chronic LBP |
| 50. | Machado LA, Kamper SJ, Herbert RD, Maher CG, McAuley JH. Analgesic effects of treatments for non-specific low back pain: a meta-analysis of placebo-controlled randomized trials. Rheumatology (Oxford). 2009;48(5):520-7. | Wrong P |
| 51. | Rajadurai V, Murugan K. Spinal manipulative therapy for low back pain: a systematic review. Phys Ther Rev. 2009;14(4):260-71. | Wrong I |
| 52. | Roine E, Roine RP, Rasanen P, Vuori I, Sintonen H, Saarto T. Cost-effectiveness of interventions based on physical exercise in the treatment of various diseases: a systematic literature review. Int J Technol Assess Health Care. 2009;25(4):427-54. | Wrong O |
| 53. | Waller B, Lambeck J, Daly D. Therapeutic aquatic exercise in the treatment of low back pain: a systematic review. Clin Rehabil. 2009;23(1):3-14. | Wrong I and C |
| 54. | Akuthota V, Ferreiro A, Moore T, Fredericson M. Core stability exercise principles. Curr Sports Med Rep. 2008;7(1):39-44. | Not SR |
| 55. | Chien JJ, Bajwa ZH. What is mechanical back pain and how best to treat it? Curr Pain Headache Rep. 2008;12(6):406-11. | Not SR |
| 56. | França FJR, Burke TN, Claret DC, Marques AP. Spinal segmental stabilisation in low-back pain: a literature review and an exercise program. Fisioterapia e Pesquisa. 2008;15(2):200-6. | Not SR |
| 57. | Hall H, McIntosh G. Low back pain (acute). BMJ Clin Evid. 2008;2008. | Not SR |
| 58. | Henchoz Y, Kai-Lik So A. Exercise and nonspecific low back pain: a literature review. Joint Bone Spine. 2008;75(5):533-9. | Not SR of RCTs |
| 59. | Silberstein N. Conquering back pain. Rehab Manag. 2008;21(7):28-9. | Not SR |
| 60. | van Duijvenbode ICD, Jellema P, van Poppel MNM, van Tulder MW. Lumbar supports for prevention and treatment of low back pain (Cochrane review) [with consumer summary]. Cochrane Database Syst Rev. 2008;Issue 2. 2008. | Wrong I and C |
| 61. | Barr KP, Griggs M, Cadby T. Lumbar stabilization: a review of core concepts and current literature, part 2. Am J Phys Med Rehabil. 2007;86(1):72-80. | Not SR |
| 62. | Chou R, Huffman LH. Nonpharmacologic therapies for acute and chronic low back pain: a review of the evidence for an American Pain Society/American College of Physicians clinical practice guideline. Ann Intern Med. 2007;147(7):492-504. | Not SR of RCTs |
| 63. | Dawson AP, McLennan SN, Schiller SD, Jull GA, Hodges PW, Stewart S. Interventions to prevent back pain and back injury in nurses: a systematic review. Occup Environ Med. 2007;64(10):642-50. | Wrong I and P |
| 64. | Kinkade S. Evaluation and treatment of acute low back pain. Am Fam Physician. 2007;75(8):1181-8. | Not SR |
| 65. | Krismer M, van Tulder M. Strategies for prevention and management of musculoskeletal conditions. Low back pain (non-specific). Best Pract Res Clin Rheumatol. 2007;21(1):77-91. | Not SR |
| 66. | Levin KH. Nonsurgical interventions for spine pain. Neurol Clin. 2007;25(2):495-505. | Not SR |
| 67. | Louw Q, Morris L, Sklaar J. Evidence of physiotherapeutic interventions for acute LBP patients. South African J Physiother. 2007;63(3):7-14. | Not SR, a guideline |
| 68. | McCamey K, Evans P. Low back pain. Prim Care. 2007;34(1):71-82. | Not SR |
| 69. | Negrini S, Minozzi S, Taricco M, Ziliani V, Zaina F. A systematic review of physical and rehabilitation medicine topics as developed by the Cochrane Collaboration. Eura Medicophys. 2007;43(3):381-90. | Not SR |
| 70. | Rand SE, Goerlich C, Marchand K, Jablecki N. The physical therapy prescription. Am Fam Physician. 2007;76(11):1661-6. | Not SR |
| 71. | Vanti C, Generali A, Ferrari S, Nava T, Tosarelli D, Pillastrini P. [ General postural rehabilitation in musculoskeletal diseases: scientific evidence and clinical indications]. Reumatismo. 2007;59(3):192-201. | Not SR |
| 72. | Bhargava A, Gelb D, Ludwig S, de Palma MJ. Physical therapy for low back pain. Current Opinion in Orthopedics. 2006 Jun;17(3):199-207. 2006. | Not SR |
| 73. | French SD, Cameron M, Walker BF, Reggars JW, Esterman AJ. A Cochrane review of superficial heat or cold for low back pain [with consumer summary]. Spine. 2006 Apr 20;31(9):998-1006. 2006. | Wrong I and C |
| 74. | Hart L. Exercise therapy for nonspecific low-back pain: a meta-analysis. Clin J Sport Med. 2006;16(2):189-90. | Not SR |
| 75. | Pittler MH, Karagulle MZ, Karagulle M, Ernst E. Spa therapy and balneotherapy for treating low back pain: meta-analysis of randomized trials. Rheumatology (Oxford). 2006;45(7):880-4. | Wrong P |
| 76. | Quinn F, Hughes C, Baxter GD. Complementary and alternative medicine in the treatment of low back pain: a systematic review. Phys Ther Rev. 2006 Jun;11(2):107-116. 2006. | Wrong P |
| 77. | Shen FH, Samartzis D, Andersson GB. Nonsurgical management of acute and chronic low back pain. J Am Acad Orthop Surg. 2006;14(8):477-87. | Not SR |
| 78. | van Tulder MW, Koes B, Malmivaara A. Outcome of non-invasive treatment modalities on back pain: an evidence-based review. Eur Spine J. 2006;15 Suppl 1:S64-81. | Not SR |
| 79. | Barr KP, Griggs M, Cadby T. Lumbar stabilization: core concepts and current literature, Part 1. Am J Phys Med Rehabil. 2005;84(6):473-80. | Not SR |
| 80. | Burton AK, Balague F, Cardon G, Eriksen HR, Henrotin Y, Lahad A, et al. How to prevent low back pain. Best Pract Res Clin Rheumatol. 2005;19(4):541-55. | Not SR |
| 81. | Cook C, Hegedus EJ, Ramey K. Physical therapy exercise intervention based on classification using the patient response method: a systematic review of the literature. J Man Manip Ther. 2005;13(3):152-62. | Wrong O |
| 82. | Kofotolis N, Sambanis M. The influence of exercise on musculoskeletal disorders of the lumbar spine. J Sports Med Phys Fitness. 2005;45(1):84-92. | Not SR |
| 83. | Moffett JK, Mannion AF. What is the value of physical therapies for back pain? Best Pract Res Clin Rheumatol. 2005;19(4):623-38. | Not SR |
| 84. | Staal JB, Rainville J, Fritz J, van Mechelen W, Pransky G. Physical exercise interventions to improve disability and return to work in low back pain: current insights and opportunities for improvement. J Occup Rehabil. 2005;15(4):491-505. | Not SR |
| 85. | van der Roer N, Goossens M, Evers S, van Tulder MW. What is the most cost-effective treatment for patients with low back pain? A systematic review. Best Pract Res Clin Rheumatol. 2005 Aug;19(4):671-684. 2005. | Wrong O |
| 86. | Tveito TH, Hysing M, Eriksen HR. Low back pain interventions at the workplace: a systematic literature review. Occup Med (Lond). 2004;54(1):3-13. | Wrong I |
| 87. | Assendelft WJ, Morton SC, Yu EI, Suttorp MJ, Shekelle PG. Spinal manipulative therapy for low back pain. A meta-analysis of effectiveness relative to other therapies. Ann Intern Med. 2003;138(11):871-81. | Withdrawn |
| 88. | Cleland J, Schulte C, Durall C. The role of therapeutic exercise in treating instability-related lumbar spine pain: a systematic review. J Back Musculoskelet Rehabil. 2002;16(2/3):105-15. | Wrong P, chronic LBP |
| 89. | Geytenbeek J. Evidence for effective hydrotherapy. Physiotherapy. 2002;88(9):514-29. | Wrong P, chronic LBP |
| 90. | Nordin M, Welser S, Campello MA, Pietrek M. Self-care techniques for acute episodes of low back pain. Best Pract Res Clin Rheumatol. 2002;16(1):89-104. | Not SR |
| 91. | Pengel HM, Maher CG, Refshauge KM. Systematic review of conservative interventions for subacute low back pain. Clin Rehabil. 2002;16(8):811-20. | Wrong P, chronic LBP |
| 92. | Quittan M. Management of back pain. Disabil Rehabil. 2002;24(8):423-34. | Not SR |
| 93. | Smith D, McMurray N, Disler P. Early intervention for acute back injury: can we finally develop an evidence-based approach? Clin Rehabil. 2002;16(1):1-11. | Not SR |
| 94. | Atlas SJ, Deyo RA. Evaluating and managing acute low back pain in the primary care setting. J Gen Intern Med. 2001;16(2):120-31. | Not SR |
| 95. | van Tulder MW. Die behandlung von ruckenschmerzen: mythen und fakten (Treatment of low back pain: myths and facts) [German]. Der Schmerz 2001 Dec;15(6):499-503. 2001. | Not SR |
| 96. | Winett RA, Carpinelli RN. Potential health-related benefits of resistance training. Prev Med. 2001;33(5):503-13. | Not SR |
| 97. | Elders LA, van der Beek AJ, Burdorf A. Return to work after sickness absence due to back disorders--a systematic review on intervention strategies. Int Arch Occup Environ Health. 2000;73(5):339-48. | Wrong O |
| 98. | Underwood MR. Exercise and the prevention of back pain disability. Br J Sports Med. 2000;34(1):5. | Not SR |
| 99. | Haigh R, Clarke AK. Effectiveness of rehabilitation for spinal pain. Clin Rehabil. 1999;13 Suppl 1:63-81. | Not SR |
| 100. | Malanga GA, Nadler SF. Nonoperative treatment of low back pain. Mayo Clin Proc. 1999;74(11):1135-48. | Not SR |
| 101. | McLain K, Powers C, Thayer P, Seymour RJ. Effectiveness of exercise versus normal activity on acute low back pain: an integrative synthesis and meta-analysis. Online J Knowl Synth Nurs. 1999;6(7):N.PAG-N.PAG. | Wrong O and S |
| 102. | Samanta A, Beardsley J. Low back pain: which is the best way forward? BMJ. 1999;318(7191):1122-3. | Not SR |
| 100. | Kuritzky L. Low back pain. Compr Ther. 1997;23(5):332-6. | No SR |
| 103. | van Poppel MN, Koes BW, Smid T, Bouter LM. A systematic review of controlled clinical trials on the prevention of back pain in industry. Occup Environ Med. 1997;54(12):841-7. | Wrong O |
| 104. | Waddell G, Feder G, Lewis M. Systematic reviews of bed rest and advice to stay active for acute low back pain. British J Gen Pract. 1997 Oct;47(423):647-652. 1997. | Not SR |
| 105. | Waddell G, Feder G, McIntosh A, Lewis M, Hutchinson A. Low back pain evidence review. J Royal College Gen Pract. 1996;1-34. 1996. | Not SR |
| 106. | Twomey L, Taylor J. Exercise and spinal manipulation in the treatment of low back pain. Spine (Phila Pa 1976). 1995;20(5):615-9. | Not SR |
| 107. | Beckerman H, Bouter LM, van der Heijden GJ, de Bie RA, Koes BW. Efficacy of physiotherapy for musculoskeletal disorders: what can we learn from research? British J Gen Pract. 1993 Feb;43(367):73-77. 1993. | Not SR |
| 108. | Tan JC, Roux EB, Dunand J, Vischer TL. Role of physical therapy in the management of common low back pain. Baillieres Clin Rheumatol. 1992;6(3):629-55. | Not SR |
| 109. | Nachemson A. Recent advances in the treatment of low back pain. Int Orthop. 1985;9(1):1-10. | Not SR |
| 110. | Rose MJ. Evaluation of the physical management of low back pain. Int Rehabil Med. 1979;1(2):83-6. | Not SR |

Abbrevations: SR=Systematic Review; LBP=Low Back Pain; P=Population; O=Outcome; I=Intervention; C=Comparison; RCT=Randomized Controlled Trial
