# Supplementary material for: Effects of exercise therapy in patients with acute low back pain: a systematic review of systematic reviews
Source: Syst Rev. 2020 Aug 14;9:182. doi: 10.1186/s13643-020-01412-8 (PMC7427286; doi:10.1186/s13643-020-01412-8)
Supplement: Supplementary file 4 — Additional file 4. Summary of Findings Tables 4-12. [file 13643_2020_1412_MOESM4_ESM.docx]

**Additional file 4.** Summary of findings Tables 4-12

Table 4. Summary of findings: Effects of general exercise therapy on pain page 2

Table 5. Summary of findings: Effects of stabilization exercise on pain page 3

Table 6. Summary of findings: Effects of McKenzie therapy on pain page 4

Table 7. Summary of findings: Effects of general exercise therapy on disability page 5

Table 8. Summary of findings: Effects of stabilization exercise on disability page 6

Table 9. Summary of findings: Effects of McKenzie therapy on disability page 7

Table 10. Summary of findings: Effects of general exercise therapy on recurrence page 8

Table 11. Summary of findings: Effects of stabilization exercise on recurrence page 9

Table 12. Summary of findings: Effects of McKenzie therapy on recurrence page 10

**Table 4.** Summary of findings: Effects of general exercise therapy on pain

| **Population**: adult patients with acute low back pain | | | | |
| --- | --- | --- | --- | --- |
| **Intervention:** general exercise therapy | | | | |
| **Comparison:** usual care, sham ultrasound, hot-pack, spinal manipulation therapy, manual therapy, stabilization exercise, ice-pack | | | | |
| **Outcome:** pain | | | | |
| **Setting:** primary care | | | | |
| **Effects of general exercise therapy on pain 0-100 (measures transformed to a 0-100 scale)** | | | | |
| **Mean** | **Mean difference (95% CI)** | **№ of participants  (studies)** | **Certainty of the evidence (GRADE)** | **Conclusion** |
| **Comparison** | **General exercise therapy** |  |  |  |
| **Post-treatment effects** | | | | |
| Usual care: 24.89 | 0.37 lower (3.55 lower to 2.81 higher) | 628 (4 RCTs) [51,59,66,75] | ⨁⨁⨁◯ MODERATE^a^ | General exercise therapy probably results in little or no difference in pain in comparison with usual care. |
| Sham ultrasound: 21.00 | 0.82 lower (6.53 lower to 4.89 higher) | 318 (1 RCT) [59] | ⨁⨁⨁◯ MODERATE^a^ | General exercise therapy probably results in little or no difference in pain in comparison with sham ultrasound. |
| Hot-pack: 22.80 | 12.6 lower (25.61 lower to 0.41 higher) | 54 (1 RCT) [75] | ⨁◯◯◯ VERY LOW^aa,b^ | It is uncertain whether general exercise therapy reduces pain in comparison with hot-pack. |
| Spinal manipulation therapy: 28.00 | 2.00 higher (12.10 lower to 16.10 higher) | 48 (1 RCT) [50] | ⨁◯◯◯ VERY LOW^a,b^ | It is uncertain whether general exercise therapy reduces pain in comparison with spinal manipulation therapy. |
| Manual therapy: not reported | 2.00 lower (8.00 lower to 4.00 higher) | 113 (1 RCT) [73] | ⨁◯◯◯ VERY LOW^a,b,c^ | It is uncertain whether general exercise therapy reduces pain in comparison with manual therapy. |
| Stabilization exercise: 31.80 | 5.1 lower (22.03 lower to 11.83 higher) | 33 (1 RCT) [77] | ⨁⨁◯◯ LOW^a,b^ | General exercise therapy may result in a small possibly unimportant effect in pain in comparison with stabilization exercise. |
| Ice-pack: 38.23 | 2.94 lower (4.73 lower to 1.15 lower) | 87 (1 RCT) [61] | ⨁⨁◯◯ LOW^a,b^ | General exercise therapy may result in a small possibly unimportant effect in pain in comparison with ice-pack. |
| **Short-term effects** | | | | |
| Sham ultrasound: 17.46 | 3.18 lower (9.94 lower to 3.58 higher) | 318 (1 RCT) [59] | ⨁⨁⨁◯ MODERATE^a^ | General exercise therapy probably results in little or no difference in pain in comparison with sham ultrasound. |
| Usual care: 12.60 | 0.50 lower (0.00 lower to 0.00 higher) | 260 (1 RCT) [59] | ⨁⨁⨁◯ MODERATE^a^ | General exercise therapy probably results in little or no difference in pain in comparison with usual care. |
| Stabilization exercise: 25.90 | 1.2 higher (15.84 lower to 18.24 higher) | 33 (1 RCT) [77] | ⨁⨁◯◯ LOW^a,b^ | General exercise therapy may result in little or no difference in pain in comparison with stabilization exercise. |
| Hot-pack: 20.10 | 12.00 lower (27.79 lower to 3.79 higher) | 54 (1 RCT) [75] | ⨁◯◯◯ VERY LOW^aa,b^ | It is uncertain whether general exercise therapy reduces pain in comparison with hot-pack. |
| Bed rest: NR | No significant difference, p<0.05 | 130 (1RCT) [52] | ⨁⨁◯◯ LOW^a,b^ | General exercise therapy may result in little or no difference in pain in comparison with bed rest. |
| Usual care/ manual therapy: NR | No significant difference, p=0.05 | 146 (1RCT) [66] | ⨁◯◯◯ VERY LOW^a,b,c^ | It is uncertain whether general exercise therapy reduces pain in comparison with usual care/manual therapy. |
| **Long-term effects** | | | | |
| Sham ultrasound: 12.74 | 0.82 lower (7.18 lower to 5.54 higher) | 318 (1 RCT) [59] | ⨁⨁⨁◯ MODERATE^a^ | General exercise therapy probably does not reduce pain in comparison with sham ultrasound. |
| Usual care: 9.60 | 0.50 higher (0.00 lower to 0.00 higher) | 271 (1 RCT) [59] | ⨁⨁⨁◯ MODERATE^a^ | General exercise therapy probably does not reduce pain in comparison with usual care. |
| Bed rest | No significant difference, p<0.05 | 130 (1 RCT) [52] | ⨁⨁◯◯ LOW^a,b^ | General exercise therapy may result in little or no difference in pain in comparison with bed rest. |
| **a.** Downgraded for risk of bias (outcome assessor not blinded); **aa.** Downgraded for very serious risk of bias (no intention to treat analysis); **b.** Downgraded for imprecision (small sample size or wide CI); **c.** Downgraded for indirectness (controls may have used the same intervention in some cases).  Data collected from Hayden 2005 [69], Rubinstein 2012 [23], Macedo 2016 [17], and Liddle 2007 [80] | | | | |
| **GRADE Working Group grades of evidence** | | | | |
| **High certainty:** We are very confident that the true effect lies close to that of the estimate of the effect | | | | |
| **Moderate certainty:** We are moderately confident in the effect estimate: The true effect is likely to be close to the estimate of the effect, but there is a possibility that it is substantially different | | | | |
| **Low certainty:** Our confidence in the effect estimate is limited: The true effect may be substantially different from the estimate of the effect | | | | |
| **Very low certainty:** We have very little confidence in the effect estimate: The true effect is likely to be substantially different from the estimate of effect | | | | |
| CI=Confidence interval; Mean=mean value in comparison group at follow-up; Mean difference=mean difference between intervention and comparison group, where ”lower” indicates less pain and “higher” indicates more pain; NR=not reported | | | | |

**Table 5.** Summary of findings: Effects of stabilization exercise on pain

| **Population**: adult patients with acute low back pain | | | | |
| --- | --- | --- | --- | --- |
| **Intervention:** stabilization exercise | | | | |
| **Comparison:** other exercise, medical management | | | | |
| **Outcome:** pain | | | | |
| **Setting:** primary care | | | | |
| **Effects of stabilization exercise on pain 0-100 (measures transformed to a 0-100 scale)** | | | | |
| **Mean** | **Mean difference (95% CI)** | **№ of participants  (studies)** | **Certainty of the evidence (GRADE)** | **Conclusion** |
| **Comparison** | **Stabilization exercise** |  |  |  |
| **Post-treatment effects** | | | | |
| Other exercise: 27.90 | 5.74 higher (3.34 lower to 14.82 higher) | 89 (2 RCTs) [77,83] | ⨁⨁⨁◯ MODERATE^a^ | Stabilization exercise probably does not reduce pain in comparison with other exercise. |
| Medical management alone: 10.40 | 9.30 lower (20.41 lower to 1.81 higher) | 41 (1 RCT) [72] | ⨁◯◯◯ VERY LOW^a,b,c^ | It is uncertain whether stabilization exercise + medical management reduces pain in comparison with medical management alone. |
| **a.** Downgraded for imprecision (small sample size); **b**. Downgraded for risk of bias (outcome assessor not blinded); **c.** Downgraded for indirectness (ultrasound of multifidi not done in clinical practice).  Data collected from Macedo 2016 [17] | | | | |
| **Short-term effects** | | | | |
| General exercise therapy: 27.10 | 1.20 lower (18.24 lower to 15.84 higher) | 33 (1 RCT) [77] | ⨁⨁◯◯ LOW^a^ | Stabilization exercise may result in little or no difference in pain in comparison with general exercise therapy. |
| **a.** Downgraded for serious imprecision (small sample size and wide CI including a possible negative effect).  Data collected from Macedo 2016 [17] | | | | |
| **GRADE Working Group grades of evidence** | | | | |
| **High certainty:** We are very confident that the true effect lies close to that of the estimate of the effect | | | | |
| **Moderate certainty:** We are moderately confident in the effect estimate: The true effect is likely to be close to the estimate of the effect, but there is a possibility that it is substantially different | | | | |
| **Low certainty:** Our confidence in the effect estimate is limited: The true effect may be substantially different from the estimate of the effect | | | | |
| **Very low certainty:** We have very little confidence in the effect estimate: The true effect is likely to be substantially different from the estimate of effect | | | | |
| CI=Confidence interval; Mean=mean value in comparison group at follow-up; Mean difference=mean difference between intervention and comparison group, where ”lower” indicates less pain and “higher” indicates more pain. | | | | |

**Table 6.** Summary of findings: Effects of McKenzie therapy on pain

| **Population**: adult patients with acute low back pain | | | | |
| --- | --- | --- | --- | --- |
| **Intervention:** McKenzie therapy | | | | |
| **Comparison:** usual care, educational booklet, spinal manipulation therapy | | | | |
| **Outcome:** pain | | | | |
| **Setting:** primary care | | | | |
| **Effects of McKenzie therapy on pain 0-100 (measures transformed to a 0-100 scale)** | | | | |
| **Mean** | **Mean difference (95% CI)** | **№ of participants  (studies)** | **Certainty of the evidence (GRADE)** | **Conclusion** |
| **Comparison** | **McKenzie Therapy** |  |  |  |
| **Post-treatment effects** | | | | |
| Usual care: 28.00 | 5.35 higher (6.91 lower to 17.61 higher) | 333 (3 RCTs) [82,85] | ⨁◯◯◯ VERY LOW^a,b,c^ | It is uncertain whether McKenzie therapy reduces pain in comparison with usual care. |
| Educational booklet: 31.00 | 11.30 lower (18.15 lower to 4.45 lower) | 240 (2 RCTs) [65,79] | ⨁⨁◯◯ LOW^a,b^ | McKenzie therapy may reduce pain slightly. |
| Spinal manipulation therapy: 25.15 | 5.76 lower (24.61 lower to 13.10 higher) | 266 (2 RCTs) [65,74] | ⨁◯◯◯ VERY LOW^a,b,c^ | It is uncertain whether McKenzie therapy reduces pain in comparison with spinal manipulation therapy. |
| **a.** Downgraded for imprecision (small sample size); **b**. Downgraded for risk of bias (uncertainty regarding the blinding process); **c.** Downgraded for inconsistency (statistical heterogeneity).  Data collected from Hayden 2005 [69], Lam 2018 [89], Dunsford 2011 [87], Rubinstein 2012 [23], and Clare 2004 [70] | | | | |
| **Short-term effects** | | | | |
| Usual care: 14.55 | 3.74 higher (2.04 lower to 9.52 higher) | 194 (2 RCTs) [60,67] | ⨁◯◯◯ VERY LOW^a,b,c^ | It is uncertain whether McKenzie therapy reduces pain in comparison with usual care. |
| Educational booklet: 27.00 | 5.00 higher (14.16 lower to 4.16 higher) | 194 (1 RCT) [65] | ⨁⨁⨁◯ MODERATE^a^ | McKenzie therapy likely results in little to no difference in pain in comparison with educational booklet. |
| Spinal manipulation therapy: 20.00 | 7.00 higher (0.60 higher to 13.40 higher) | 235 (1 RCT) [65] | ⨁⨁⨁◯ MODERATE^a^ | McKenzie therapy probably does not reduce pain in comparison with spinal manipulation. |
| NSAID | No significant difference, p=0.125 | 175 (1 RCT) [54] | ⨁◯◯◯ VERY LOW^a,b,d^ | It is uncertain whether McKenzie therapy reduces pain in comparison with NSAID. |
| **a.** Downgraded for imprecision (small sample size); **b**. Downgraded for risk of bias (uncertainty regarding the blinding process; **c.** Downgraded for indirectness (different approach of McKenzie therapy; **d.** Downgraded for imprecision (no standard deviation or CI).  Data collected from Hayden 2005 [69], Rubinstein 2012 [23], and Clare 2004 [70] | | | | |
| **Intermediate-term effects** | | | | |
| NSAID | No significant difference, F=0.60 | 175 (1 RCT) [54] | ⨁◯◯◯ VERY LOW^a,b,c^ | It is uncertain whether McKenzie therapy reduces pain in comparison with NSAID |
| **a.** Downgraded for imprecision (small sample size); **b**. Downgraded for risk of bias (uncertainty regarding the blinding process); **c.** Downgraded for imprecision (no standard deviation or CI). Data collected from Roberts 1990 [54] included in Clare 2004 [70]. | | | | |
| **Long-term effects** | | | | |
| Usual care: 13.70 | 5.10 lower (19.53 lower to 9.33 higher) | 75 (1 RCT) [67] | ⨁◯◯◯ VERY LOW^a,b,c^ | It is uncertain whether McKenzie therapy reduces pain in comparison with usual care. |
| Educational booklet: 20.00 | 8.00 lower (14.37 lower to 1.63 lower) | 194 (1 RCT) [65] | ⨁⨁⨁◯ MODERATE^a^ | McKenzie therapy likely results in a small possibly unimportant effect in pain in comparison with educational booklet. |
| Spinal manipulation therapy | No significant difference, p=0.16 | 231 (1 RCT) [65] | ⨁⨁⨁◯ MODERATE^a^ | McKenzie therapy probably result in little or no difference on pain in comparison with spinal manipulation. |
| Mini back school | Significant difference in favour of McKenzie therapy | 95 (1 RCT) [53] | ⨁◯◯◯ VERY LOW^a,b,c,d^ | It is uncertain whether McKenzie therapy reduces pain in comparison with mini back school. |
| NSAID | No significant difference | 175 (1 RCT) [54] | ⨁⨁◯◯ LOW^a,b,d^ | McKenzie therapy may result in little or no difference in pain in comparison with NSAID. |
| **a.** Downgraded for imprecision (small sample size); **b**. Downgraded for risk of bias (uncertainty regarding the blinding process); **c.** Downgraded for indirectness (different approach of McKenzie therapy; **d.** Downgraded for imprecision (no standard deviation or CI). Data collected from Hayden 2005 [69], Koes 1991 [49], Clare 2004 [70]. | | | | |
| **GRADE Working Group grades of evidence** | | | | |
| **High certainty:** We are very confident that the true effect lies close to that of the estimate of the effect | | | | |
| **Moderate certainty:** We are moderately confident in the effect estimate: The true effect is likely to be close to the estimate of the effect, but there is a possibility that it is substantially different | | | | |
| **Low certainty:** Our confidence in the effect estimate is limited: The true effect may be substantially different from the estimate of the effect | | | | |
| **Very low certainty:** We have very little confidence in the effect estimate: The true effect is likely to be substantially different from the estimate of effect | | | | |
| CI=Confidence interval; Mean=mean value in comparison group at follow-up; Mean difference=mean difference between intervention and comparison group, where ”lower” indicates less pain and “higher” indicates more pain. | | | | |

**Table 7.** Summary of findings: Effects of general exercise therapy on disability

| **Population**: adult patients with acute low back pain | | | | |
| --- | --- | --- | --- | --- |
| **Intervention:** general exercise therapy | | | | |
| **Comparison:** usual care, sham ultrasound, spinal manipulation therapy, hot-pack, NSAID, bed rest | | | | |
| **Outcome:** disability | | | | |
| **Setting:** primary care | | | | |
| **Effects of general exercise therapy on disability 0-100 (measures transformed to a 0-100 scale)** | | | | |
| **Mean** | **Mean difference (95% CI)** | **№ of participants  (studies)** | **Certainty of the evidence (GRADE)** | **Conclusion** |
| **Comparison** | **General exercise therapy** |  |  |  |
| **Post-treatment effects** | | | | |
| Usual care: 19.88 | 2.62 higher (0.52 higher to 4.72 higher) | 530 (3 RCTs) [52, 59,66] | ⨁⨁⨁◯ MODERATE^b^ | General exercise therapy likely does not reduce disability in comparison with usual care. |
| Sham ultrasound: 15.30 | 2.00 higher (2.07 lower to 6.07 higher) | 318 (1 RCT) [59] | ⨁⨁⨁◯ MODERATE^a^ | General exercise therapy likely does not reduce disability in comparison with sham ultrasound. |
| Spinal manipulation therapy: 40.00 | 3.34 higher (5.08 lower to 11.76 higher) | 48 (1 RCT) [50] | ⨁⨁◯◯ LOW^a,b^ | General exercise may result in little or no difference in disability in comparison with spinal manipulation. |
| Hot-pack: 39.62 | 22.94 lower (34.59 lower to 11.29 higher) | 54 (1 RCT) [75] | ⨁◯◯◯ VERY LOW^a,b,c^ | It is uncertain whether general exercise therapy reduces disability in comparison with hot-pack. |
| NSAID | No significant difference | 70 (1 RCT) [66] | ⨁◯◯◯ VERY LOW^a,b,c^ | It is uncertain whether general exercise therapy reduces disability in comparison with NSAID. |
| **a.** Downgraded for imprecision (small sample size); **b**. Downgraded for risk of bias (uncertainty regarding the blinding process); **c.** Downgraded for imprecision (no standard deviation or CI). Data collected from Hayden 2005 [69] and van Tulder 2000 [64] | | | | |
| **Short-term effects** | | | | |
| Usual care: 12.65 | 0.84 higher (2.00 lower to 3.67 higher) | 357 (2 RCTs) [59,66] | ⨁⨁⨁◯ MODERATE^b^ | General exercise therapy likely does not reduce disability in comparison with usual care. |
| Sham ultrasound: 10.30 | 2.00 higher (2.31 lower to 6.31 higher) | 316 (1 RCT) [59] | ⨁⨁⨁◯ MODERATE^a^ | General exercise therapy likely does not reduce disability in comparison with sham ultrasound. |
| Hot-pack: 16.68 | 4.17 lower (16.66 lower to 8.32 higher) | 54 (1 RCT) [75] | ⨁◯◯◯ VERY LOW^a,b,c^ | It is uncertain whether general exercise therapy reduces disability in comparison with hot-pack. |
| Bed rest | No significant difference | 125 (1 RCT) [52] | ⨁◯◯◯ VERY LOW^a,b,c^ | It is uncertain whether general exercise therapy reduces disability in comparison with bed rest. |
| **a.** Downgraded for imprecision (small sample size); **b**. Downgraded for risk of bias (uncertainty regarding the blinding process); **c.** Downgraded for imprecision (wide CI or no CI). Data collected from Hayden 2005 [69] and van Tulder 2000 [64] | | | | |
| **Long-term effects** | | | | |
| Usual care: 9.65 | 0.57 lower (3.36 lower to 2.23 higher) | 354 (2 RCTs) [59,66] | ⨁⨁◯◯ LOW^a,b,c^ | General exercise therapy may result in little or no difference in disability in comparison with usual care. |
| Sham ultrasound: 8.30 | 2.00 higher (2.19 lower to 6.19 higher) | 316 (1 RCT) [59] | ⨁⨁⨁◯ MODERATE^a^ | General exercise therapy likely does not reduce disability in comparison with sham ultrasound. |
| Bed rest: | No significant difference | 125 (1 RCT) [52] | ⨁◯◯◯ VERY LOW^a,b,d^ | It is uncertain whether general exercise therapy reduces disability in comparison with bed rest. |
| **a.** Downgraded for imprecision (small sample size); **b**. Downgraded for risk of bias (uncertainty regarding the blinding process); **c.** Downgraded for indirectness (co-interventions similar exercise therapy as comparator); **d.** Downgraded for imprecision (no CI). Data collected from Hayden 2005 [69] and van Tulder 2000 [64] | | | | |
| **GRADE Working Group grades of evidence** | | | | |
| **High certainty:** We are very confident that the true effect lies close to that of the estimate of the effect | | | | |
| **Moderate certainty:** We are moderately confident in the effect estimate: The true effect is likely to be close to the estimate of the effect, but there is a possibility that it is substantially different | | | | |
| **Low certainty:** Our confidence in the effect estimate is limited: The true effect may be substantially different from the estimate of the effect | | | | |
| **Very low certainty:** We have very little confidence in the effect estimate: The true effect is likely to be substantially different from the estimate of effect | | | | |
| CI=Confidence interval; Mean=mean value in comparison group at follow-up; Mean difference=mean difference between intervention and comparison group, where ”lower” indicates less disability and “higher” indicates more disability. | | | | |

**Table 8.** Summary of findings: Effects of stabilization exercise on disability

| **Population**: adult patients with acute low back pain | | | | |
| --- | --- | --- | --- | --- |
| **Intervention:** stabilization exercise | | | | |
| **Comparison:** general exercise therapy, McKenzie therapy, medical management, spinal manipulation therapy | | | | |
| **Outcome:** disability | | | | |
| **Setting:** primary care | | | | |
| **Effects of stabilization exercise on disability 0-100 (measures transformed to a 0-100 scale)** | | | | |
| **Mean** | **Mean difference (95% CI)** | **№ of participants  (studies)** | **Certainty of the evidence (GRADE)** | **Conclusion** |
| **Comparison** | **Stabilization exercise** |  |  |  |
| **Post-treatment effects** | | | | |
| General exercise therapy: 39.20 | 8.40 lower (24.33 lower to 7.53 higher) | 33 (1 RCT) [77] | ⨁◯◯◯ VERY LOW^a,b,c^ | It is uncertain whether stabilization exercise reduces disability in comparison with general exercise therapy. |
| McKenzie therapy: 20.60 | 1.30 higher (5.90 lower to 8.50 higher) | 83 (1 RCT) [83] | ⨁⨁◯◯ LOW^a,b^ | Stabilization exercise may result in little or no difference in disability in comparison with McKenzie therapy. |
| Medical management alone: not reported | 0.90 lower (4.77 lower to 2.97 higher) | 41 (1 RCT) [72] | ⨁◯◯◯ VERY LOW^a,b,d^ | It is uncertain whether stabilization exercise + medical management reduces disability in comparison with medical management alone. |
| Spinal manipulation therapy: 17.90 | 4.00 higher (3.38 lower to 11.38 higher) | 85 (1 RCT) [83] | ⨁⨁◯◯ LOW^a,b^ | Stabilization exercise may result in little or no difference in disability in comparison with spinal manipulation. |
| **a.** Downgraded for imprecision (small sample size); **b**. Downgraded for risk of bias (uncertainty regarding the blinding process); Downgraded for imprecision (wide CI); **d.** Downgraded for indirectness (not possible to rule out asymmetrical difference of multifidus in clinical practice). Data collected from Macedo 2016 [17] | | | | |
| **Short-term effects** | | | | |
| General exercise therapy: 28.30 | 6.70 lower (22.80 lower to 9.40 higher) | 33 (1 RCT) [77] | ⨁⨁◯◯ LOW^a,b^ | Stabilization exercise may result in little or no difference in disability in comparison with general exercise therapy. |
| **a.** Downgraded for imprecision (small sample size); **b**. Downgraded for risk of bias (uncertainty regarding the blinding process). Data collected from Macedo 2016 [17] | | | | |
| **Long-term effects** | | | | |
| McKenzie therapy: 14.80 | 5.70 higher (1.38 lower to 12.78 higher) | 83 (1 RCT) [83] | ⨁⨁◯◯ LOW^a,b^ | Stabilization exercise may result in little or no difference in disability in comparison with McKenzie therapy. |
| Spinal manipulation therapy: 16.80 | 3.70 higher (4.10 lower to 11.50 higher) | 85 (1 RCT) [83] | ⨁⨁◯◯ LOW^a,b^ | Stabilization exercise may result in little or no difference in disability in comparison with spinal manipulation therapy. |
| **a.** Downgraded for imprecision (small sample size); **b**. Downgraded for risk of bias (uncertainty regarding the blinding process). Data collected from Macedo 2016 [17] | | | | |
| **GRADE Working Group grades of evidence** | | | | |
| **High certainty:** We are very confident that the true effect lies close to that of the estimate of the effect | | | | |
| **Moderate certainty:** We are moderately confident in the effect estimate: The true effect is likely to be close to the estimate of the effect, but there is a possibility that it is substantially different | | | | |
| **Low certainty:** Our confidence in the effect estimate is limited: The true effect may be substantially different from the estimate of the effect | | | | |
| **Very low certainty:** We have very little confidence in the effect estimate: The true effect is likely to be substantially different from the estimate of effect | | | | |
| CI=Confidence interval; Mean=mean value in comparison group at follow-up; Mean difference=mean difference between intervention and comparison group, where ”lower” indicates less disability and “higher” indicates more disability. | | | | |

**Table 9.** Summary of findings: Effects of McKenzie therapy on disability

| **Population**: adult patients with acute low back pain | | | | |
| --- | --- | --- | --- | --- |
| **Intervention:** McKenzie therapy | | | | |
| **Comparison:** usual care, educational booklet, spinal manipulation therapy, NSAID | | | | |
| **Outcome:** disability | | | | |
| **Setting:** primary care | | | | |
| **Effects of McKenzie therapy on disability 0-100 (measures transformed to a 0-100 scale)** | | | | |
| **Mean** | **Mean difference (95% CI)** | **№ of participants  (studies)** | **Certainty of the evidence (GRADE)** | **Conclusion** |
| **Comparison** | **McKenzie therapy** |  |  |  |
| **Post-treatment effects** | | | | |
| Usual care: 20.40 | 3.27 higher (5.93 lower to 12.48 higher) | 332 (3 RCTs) [27,60,67] | ⨁⨁◯◯ LOW^a,b^ | McKenzie therapy may result in little or no difference in disability in comparison with  usual care. |
| Educational booklet: 23.70 | 3.74 lower (8.75 lower to 1.26 higher) | 243 (2 RCTs) [65,79] | ⨁⨁◯◯ LOW^a,b^ | McKenzie therapy may result in little or no difference in disability in comparison with  educational booklet. |
| Spinal manipulation therapy: 22.98 | 1.32 lower (5.84 lower to 3.21 higher) | 323 (3 RCTs) [16,67,83] | ⨁⨁⨁◯ MODERATE^a^ | McKenzie therapy likely results in little to no difference in disability in comparison with spinal manipulation therapy. |
| **a.** Downgraded for imprecision (small sample size); **b**. Downgraded for risk of bias (uncertainty regarding the blinding process). Data collected from Hayden 2005 [69] | | | | |
| **Short-term effects** | | | | |
| Usual care: 7.75 | 2.19 higher (2.37 lower to 6.76 higher) | 194 (2 RCTs) [60,67] | ⨁⨁◯◯ LOW^a,b^ | McKenzie therapy appears to not reduce disability in comparison with usual care. |
| Educational booklet: 18.70 | 0.86 lower (7.26 lower to 5.54 higher) | 194 (1 RCT) [65] | ⨁⨁⨁◯ MODERATE^a^ | McKenzie therapy likely results in little to no difference in disability in comparison with educational booklet. |
| Spinal Manipulation: 31 | 2.2 higher (0.3 lower to 4.8 higher) | 235 (1 RCT) [65] | ⨁⨁⨁◯ MODERATE^a^ | McKenzie therapy likely results in little to no difference in disability in comparison with spinal manipulation therapy. |
| NSAID: 4 | 4.17 lower (9.44 lower to 1.10 higher) | 195 (1 RCT) [54] | ⨁⨁◯◯ LOW^a,b^ | McKenzie therapy may result in little or no difference in disability in comparison with  NSAID. |
| **a.** Downgraded for imprecision (small sample size); **b**. Downgraded for risk of bias (uncertainty regarding the blinding process). Data collected from Hayden 2005 [69], Clare 2004 [70], Rubinstein 2012 [23] | | | | |
| **Intermediate-term effects** | | | | |
| Educational booklet: not reported | 0.90 lower (7.60 lower to 5.80 higher) | 177 (1 RCT) [65] | ⨁⨁⨁◯ MODERATE^a^ | McKenzie therapy likely results in little to no difference in disability in comparison with educational booklet. |
| NSAID | No significant difference, F=0.99 | 175 (1 RCT) [54] | ⨁◯◯◯ VERY LOW^a,b,c^ | It is uncertain whether McKenzie therapy reduces pain in comparison with NSAID. |
| **a.** Downgraded for imprecision (small sample size); **b**. Downgraded for risk of bias (uncertainty regarding the blinding process); **c.** Downgraded for imprecision (no CI). Data collected from Hayden 2005 [69], Clare 2004 [70] | | | | |
| **Long-term effects** | | | | |
| Usual care: 8.00 | 3.30 lower (12.55 lower to 5.95 higher) | 75 (1 RCT) [67] | ⨁◯◯◯ VERY LOW^a,b,c^ | It is uncertain whether McKenzie therapy reduces disability in comparison with usual care. |
| Educational booklet: 11.31 | 8.27 lower (13.89 lower to 2.65 higher) | 194 (1 RCT) [65] | ⨁⨁⨁◯ MODERATE^a^ | McKenzie therapy likely results in a small possibly unimportant effect in disability in comparison with educational booklet. |
| Spinal manipulation therapy: 16.80 | 0.12 higher (0.42 lower to 0.67 higher) | 57 (1 RCT) [83] | ⨁◯◯◯ VERY LOW^a,b,c^ | It is uncertain whether McKenzie therapy reduces disability in comparison with spinal manipulation therapy. |
| NSAID | No significant difference, F=0.60 | 141 (1 RCT) [54] | ⨁◯◯◯ VERY LOW^a,b,c^ | It is uncertain whether McKenzie therapy reduces pain in comparison with NSAID. |
| **a.** Downgraded for imprecision (small sample size); **b**. Downgraded for risk of bias (uncertainty regarding the blinding process); **c.** Downgraded for imprecision (wide or no CI). Data collected from Hayden 2005 [69], Rubinstein 2012 [23], Clare 2004 [70] | | | | |
| **GRADE Working Group grades of evidence** | | | | |
| **High certainty:** We are very confident that the true effect lies close to that of the estimate of the effect | | | | |
| **Moderate certainty:** We are moderately confident in the effect estimate: The true effect is likely to be close to the estimate of the effect, but there is a possibility that it is substantially different | | | | |
| **Low certainty:** Our confidence in the effect estimate is limited: The true effect may be substantially different from the estimate of the effect | | | | |
| **Very low certainty:** We have very little confidence in the effect estimate: The true effect is likely to be substantially different from the estimate of effect | | | | |
| CI=Confidence interval; Mean=mean value in comparison group at follow-up; Mean difference=mean difference between intervention and comparison group, where ”lower” indicates less disability and “higher” indicates more disability. | | | | |

**Table 10.** Summary of findings: Effects of general exercise therapy on recurrence

| **Population**: adult patients with acute low back pain  **Intervention:** general exercise therapy  **Comparison:** sham ultrasound, usual care, ice-pack  **Outcome:** recurrence  **Setting:** primary care | | | | | |
| --- | --- | --- | --- | --- | --- |
| **Effects of general exercise therapy on recurrence** | | | | | |
| **Anticipated absolute effects* (95% CI)** | | **Relative effect (95% CI)** | **№ of participants  (studies)** | **Certainty of the evidence (GRADE)** | **Conclusion** |
| **Risk with comparison** | **Risk with General exercise therapy** |  |  |  |  |
| **Long-term effects** | | | | | |
| Sham ultrasound: 660 per 1000 | 694 per 1000 (594 to 806) | RR 1.05 (0.90 to 1.22) | 316 (1 RCT) [59] | ⨁⨁⨁◯ MODERATE^a^ | General exercise therapy probably does not reduce recurrence more than sham ultrasound. |
| Usual care: 697 per 1000 | 697 per 1000 (599 to 808) | RR 1.00 (0.86 to 1.16) | 309 (1 RCT) [59] | ⨁⨁⨁◯ MODERATE^a^ | General exercise therapy probably does not reduce recurrence more than usual care. |
| Ice-pack: 609 per 1000 | 651 per 1000 (444 to 962) | RR 1.07 (0.73 to 1.58) | 72 (1 RCT) [61] | ⨁◯◯◯ VERY LOW^a,b,c^ | It is uncertain whether general exercise therapy reduces recurrence compared with ice-pack. |
| **a.** Downgraded for imprecision (small sample size). **b**. Downgraded for risk of bias (uncertainty regarding the blinding process); **c.** Downgraded for imprecision (wide CI). Data collected from Choi 2010 [85], Machado 2006 [76] | | | | | |
| ***The risk in the intervention group** (and its 95% CI) is based on the assumed risk in the comparison group and the **relative effect** of the intervention (and its 95% CI). | | | | | |
| **GRADE Working Group grades of evidence**  **High certainty:** We are very confident that the true effect lies close to that of the estimate of the effect  **Moderate certainty:** We are moderately confident in the effect estimate: The true effect is likely to be close to the estimate of the effect, but there is a possibility that it is substantially different  **Low certainty:** Our confidence in the effect estimate is limited: The true effect may be substantially different from the estimate of the effect  **Very low certainty:** We have very little confidence in the effect estimate: The true effect is likely to be substantially different from the estimate of effect | | | | | |
| CI=Confidence interval; RR= Risk ratio. | | | | | |

**Table 11.** Summary of findings: Effects of stabilization exercise on recurrence

| **Population**: adult patients with acute low back pain  **Intervention:** stabilization exercise + medical management  **Comparison:** medical management alone  **Outcome:** recurrence, recurrence frequency  **Setting:** primary care | | | | | |
| --- | --- | --- | --- | --- | --- |
| **Effects of stabilization exercise on recurrence** | | | | | |
| **Anticipated absolute effects* (95% CI)** | | **Relative effect (95% CI)** | **№ of participants  (studies)** | **Certainty of the evidence (GRADE)** | **Conclusion** |
| **Risk with comparison** | **Risk with stabilization exercise** |  |  |  |  |
| **Long-term effects** | | | | | |
| Medical management: 842 per 1000 | 320 per 1000 (152 to 606)) | RR 0.36 (0.18 to 0.72) | 39 (1 RCT) [72] | ⨁◯◯◯ VERY LOW^a,b^ | It is uncertain whether stabilization exercise + medical management reduces recurrence in comparison with medical management alone. |
| **Mean** | **Mean difference (95% CI)** | - | 39 (1 RCT) [72] | ⨁◯◯◯ VERY LOW^a,b^ | It is uncertain whether stabilization exercise + medical management reduces recurrence frequency in comparison with medical management alone. |
| Medical management: 4.20 | 1.40 fewer (3.16 fewer to 0.36 more) |  |  |  |  |
| **a.** Downgraded for serious imprecision (small sample size and wide CI); **b**. Downgraded for risk of bias (uncertainty regarding the blinding process). Data collected from Macedo 2016 [17] | | | | | |
| ***The risk in the intervention group** (and its 95% CI) is based on the assumed risk in the comparison group and the **relative effect** of the intervention (and its 95% CI). | | | | | |
| **GRADE Working Group grades of evidence**  **High certainty:** We are very confident that the true effect lies close to that of the estimate of the effect  **Moderate certainty:** We are moderately confident in the effect estimate: The true effect is likely to be close to the estimate of the effect, but there is a possibility that it is substantially different  **Low certainty:** Our confidence in the effect estimate is limited: The true effect may be substantially different from the estimate of the effect  **Very low certainty:** We have very little confidence in the effect estimate: The true effect is likely to be substantially different from the estimate of effect | | | | | |
| CI=Confidence interval. RR= Risk ratio; Mean=mean value in comparison group at follow-up; Mean difference=mean difference between intervention and comparison group. | | | | | |

**Table 12**. Summary of findings: Effects of McKenzie therapy on recurrence

| **Population**: adult patients with acute low back pain  **Intervention:** McKenzie therapy  **Comparison:** NSAID, minimal education  **Outcome:** recurrence, recurrence frequency  **Setting:** primary care | | | | | |
| --- | --- | --- | --- | --- | --- |
| **Effects of McKenzie therapy on recurrence** | | | | | |
| **Anticipated absolute effects* (95% CI)** | | **Relative effect (95% CI)** | **№ of participants  (studies)** | **Certainty of the evidence (GRADE)** | **Conclusion** |
| **Risk with comparison** | **Risk with McKenzie therapy** |  |  |  |  |
| **Intermediate-term effects** | | | | | |
| **Mean** | **Mean difference (95% CI)** | - | 175 (1 RCT) [54] | ⨁◯◯◯ VERY LOW^a,b^ | McKenzie therapy may result in little or no difference on recurrence in comparison with NSAID. |
| NSAID | No significant difference, p=0.18 |  |  |  |  |
| **Long-term effects** | | | | | |
| Minimal education: 625 per 1000 | 469 per 1000 (263 to 844) | RR 0.75 (0.42 to 1.35) | 294 (2 RCTs) [53,65] | ⨁◯◯◯ VERY LOW^a,b^ | It is uncertain whether McKenzie therapy reduces recurrence in comparison with minimal education. |
| **Mean** | **Mean difference (95% CI)** | - | 147 (1 RCT) [54] | ⨁◯◯◯ VERY LOW^a,b^ | It is uncertain whether McKenzie therapy reduces recurrence frequency in comparison with NSAID. |
| NSAID | No significant difference, p=0.25 |  |  |  |  |
| **a.** Downgraded for serious imprecision (small sample size or CIs do not cross each other or are absent); **b**. Downgraded for risk of bias (uncertainty regarding the blinding process). Data collected from Clare 2004 [70], Choi 2010 [85].  *The risk in the intervention group (and its 95% CI) is based on the assumed risk in the comparison group and the relative effect of the intervention (and its 95% CI). | | | | | |
| **GRADE Working Group grades of evidence**  **High certainty:** We are very confident that the true effect lies close to that of the estimate of the effect  **Moderate certainty:** We are moderately confident in the effect estimate: The true effect is likely to be close to the estimate of the effect, but there is a possibility that it is substantially different  **Low certainty:** Our confidence in the effect estimate is limited: The true effect may be substantially different from the estimate of the effect  **Very low certainty:** We have very little confidence in the effect estimate: The true effect is likely to be substantially different from the estimate of effect | | | | | |
| CI=Confidence interval; RR= Risk ratio; Mean=mean value in comparison group at follow-up; Mean difference=mean difference between intervention and comparison group. | | | | | |
